# Signaling Under Stress: Targeting the Glucocorticoid Receptor in Cancer

**DOI:** 10.1158/0008-5472.CAN-25-4426

**Published:** 2026-05-18

**Authors:** Melanie S. Flint, David M. O’Malley, Domenica Lorusso, Susan K. Lutgendorf, Alexander B. Olawaiye, Adrian M. Jubb, Premal H. Thaker

**Affiliations:** 1 School of Applied Sciences, University of Brighton, Brighton, United Kingdom.; 2 James Comprehensive Cancer Center, The Ohio State University, Columbus, Ohio.; 3 Department of Biomedical Science, Humanitas University, Milan, Italy.; 4 Gynaecology Oncology Unit, Humanitas San Pio X, Milan, Italy.; 5 Department of Psychological and Brain Sciences, University of Iowa, Iowa City, Iowa.; 6 Magee Women’s Hospital of UPMC, Pittsburgh, Pennsylvania.; 7 Corcept Therapeutics Inc., Redwood City, California.; 8 Washington University School of Medicine, St. Louis, Missouri.

## Abstract

Glucocorticoid receptor (GR) signaling is critical to our physiology but is dysregulated in cancer by the pain, psychologic distress, iatrogenic morbidity, and pathology associated with an advanced, invasive disease. Many tumors exploit glucocorticoid signaling to provide antiapoptotic survival signals, stimulate growth and metastasis, suppress immune surveillance, and drive treatment resistance. Endogenous cortisol and exogenous glucocorticoids are typically associated with poor responses to cytotoxic chemotherapy, targeted therapy, and immunotherapy in patients with solid tumors. Translational and nonclinical data show that selective GR antagonists (SGRA) synergize with anticancer agents, including taxanes, androgen receptor inhibitors, PARP inhibitors, and anti–programmed cell death ligand 1 (PDL-1) immune checkpoint inhibitors, driving improved anticancer activity. In a tumor-intrinsic manner, SGRAs downregulate the antiapoptotic proteins SGK1 and DUSP1, which induce resistance to cytotoxic chemotherapy. This synergy has been confirmed in several randomized controlled trials, which showed improved efficacy when SGRAs were added to standard-of-care taxane therapy in platinum-resistant ovarian cancer. In a distinct cellular context, SGRAs inhibit resistance to antiandrogen therapy mediated through the GR and have the potential for additive anticancer activity in prostate cancer. Herein, we summarize the role of glucocorticoid signaling in solid tumor biology, providing mechanistic insights into recent clinical advances and emphasizing key outstanding translational and clinical questions.

## Introduction

Advanced cancer causes tissue damage, pain, and psychosocial distress related to diagnosis, recurrence, and associated mortality. Patients often have limited treatment options: invasive surgery, radiotherapy, chemotherapy, targeted therapy, and immunotherapy of modest efficacy and often substantial toxicity. The disease and the treatment journey often substantially diminish daily functioning and quality of life (1, 2). The illness and its treatment cause chronic stress, a complex physiologic process that results from the integration of sensory inputs, cognitive processing, physiologic signaling, and pattern recognition in the limbic system of the brain. In a landmark randomized controlled trial, Spiegel and colleagues (3) showed that 1 year of weekly supportive group therapy, with self-hypnosis for pain, prolonged survival in women with metastatic breast cancer. Similar findings were observed in several subsequent trials (4, 5) and meta-analyses with related interventions (6, 7). Furthermore, use of exogenous corticosteroids was associated with increased metastasis in observational studies on patients with breast cancer in the 1950s and 1960s (8, 9). However, these clinical observations outpaced our mechanistic understanding of how chronic stress, steroid use, and psychosocial interventions affect cancer outcomes. Herein, we make a case for a central role for cortisol, the principal stress hormone, and the glucocorticoid receptor (GR), a key mediator of cortisol signaling, in cancer progression and the efficacy of cancer treatment. GR agonists, referred to as glucocorticoids or corticosteroids, have an established role in the treatment of lymphoid malignancies (10) that is distinct from solid tumors and has been extensively reviewed elsewhere (11, 12). In brief, GR activation in lymphoid cancers triggers lineage-specific transcriptional programs that induce potent apoptotic responses. By contrast, in solid tumors, GR signaling exerts antiapoptotic and immunosuppressive effects. The functional heterogeneity of the GR pathway in lymphoid cancers versus solid tumors and its clinical implications are summarized in Supplementary Table S1. This review will focus on solid tumor biology and the potential for drugs that selectively target the GR to enhance the efficacy of agents targeted against related nuclear hormone receptors, cytotoxic chemotherapy, and immunotherapy.

## Physiologic GR Signaling and Function

GR signaling is a central physiologic mechanism that integrates endocrine cues to regulate metabolism, immunity, and stress responses. Glucocorticoids are a class of natural and synthetic steroid molecules that agonize the GR encoded by *NR3C1*. In humans, cortisol is the endogenous glucocorticoid secreted by the adrenal gland, under the control of pituitary adrenocorticotropic hormone (ACTH) in the hypothalamic–pituitary–adrenal (HPA) axis. Under physiologic conditions, glucocorticoids regulate a wide variety of genes and cellular processes to maintain metabolic, immune, cardiovascular, renal, and bone homeostasis (13–15). In addition, cortisol influences the central nervous system, affecting mood, behavior, and sleep–wake cycles (14).

The body’s circadian rhythm is controlled by the hypothalamic suprachiasmatic nucleus (SCN), which is influenced by light/dark cycles. In the morning, the SCN signals the hypothalamus to release corticotropin-releasing hormone (CRH), which in turn stimulates the release of ACTH from the anterior pituitary gland. ACTH acts on the adrenal cortex to stimulate the synthesis and release of cortisol. As cortisol rises, it exhibits negative feedback on CRH and ACTH release, and cortisol levels decline to reach their lowest point in the late evening before sleep (14, 16). Cortisol is also released in response to physical or psychologic stress (14). Neurocognitive networks work together to regulate cognitive executive functions and responses to stimuli (17). The prefrontal cortex mediates response inhibition, a core function governing impulse control. Human studies have shown that whereas acute increases in cortisol due to stress can temporarily enhance response inhibition (18), chronically elevated cortisol due to chronic stress impairs this critical cognitive process (19). The amygdala synthesizes inputs from external senses and internal physiologic signals (e.g., hormone levels), identifying a state of stress through a complex process of pattern recognition. Signaling through the stria terminalis, the amygdala initiates CRH release by activating the hypothalamic paraventricular nucleus. The stria terminalis and noradrenaline also stimulate CRH release by activating neurons in the basal nucleus of the stria terminalis (16, 20). The consequent increase in cortisol triggers a wide variety of physiologic effects to help the body cope with stress, including increasing blood glucose levels, mobilizing fat and protein to provide additional energy, suppressing the immune system and inflammation, increasing cardiovascular responsiveness to epinephrine and norepinephrine, and increasing cognitive alertness (16, 20).

Cortisol may become dysregulated with increased endogenous secretion in states of chronic psychologic stress and chronic disease, adversely affecting multiple organ systems (13, 21, 22). Psychologically this may manifest with increased anxiety, depressed mood, irritability, emotional lability, reduced motivation, and anhedonia (21, 23). High cortisol levels cause metabolic dysregulation resulting in insulin-resistant diabetes, visceral fat deposition/redistribution, and muscle atrophy (24, 25). Patients may exhibit hypertension, water/electrolyte imbalance, osteoporosis, cataracts, peptic ulcers, and thinning of the skin (22). These effects are also known toxicities of prolonged high doses of systemically administered synthetic glucocorticoids (e.g., dexamethasone, betamethasone, and prednisone), widely used to treat inflammatory and immune diseases (26, 27).

**Figure 1.**
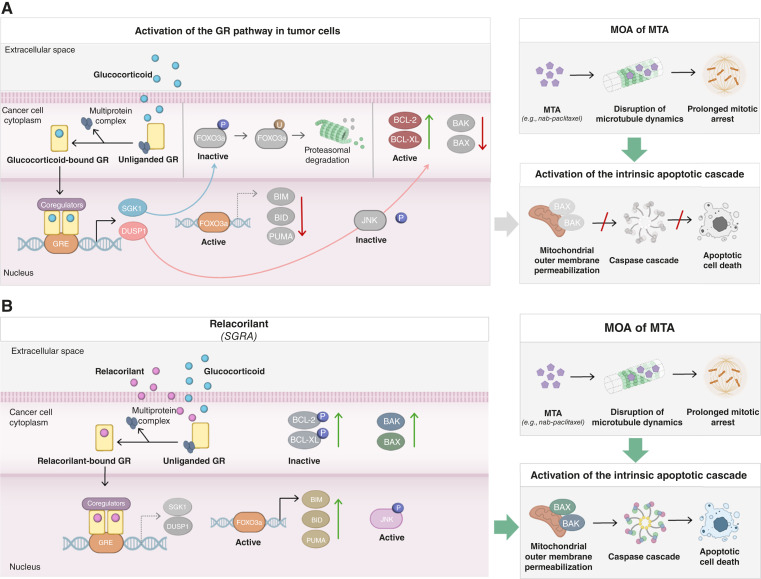
Potential mechanism of GR-mediated effects on the apoptotic cascade. **A,** Ligand activation of GR leads to its nuclear translocation, where it interacts with GREs and coregulators and upregulates antiapoptotic genes *SGK1* and *DUSP1* (30). SGK1 phosphorylates the Forkhead family transcription factor FOXO3a, resulting in its exit from the nucleus and degradation, and reduced expression of proapoptotic genes *BIM*, *BID*, and *PUMA* (31, 32). This can reduce the activation of proapoptotic effectors BAK/BAX and inhibit apoptosis. DUSP1-mediated dephosphorylation of phospho-JNK, a member of the MAP-kinase superfamily, blocks inactivation of antiapoptotic proteins BCL-2 and BCL-XL (31, 33). This may inhibit BAK/BAX activation and apoptosis. Microtubule-targeting agents induce apoptosis through disruption of microtubule dynamics and prolonged mitotic arrest (34–36). **B,** Relacorilant, through GR antagonism, downregulates *SGK1* and *DUSP1*, potentially improving MTA-induced activation of the intrinsic apoptotic cascade (37). Relacorilant (CORT125134, Corcept Therapeutics, Inc.) is an investigational, orally administered SGRA that can reverse the effects of glucocorticoids by restoring chemotherapy activity and antagonizing the antiapoptotic effects of glucocorticoids (37, 38). BAK, BCL-2 antagonist/killer; BAX, BCL-2–associated X; BCL-2, apoptosis regulator; BCL-XL, BCL-2–like 1; BID, BH3 interacting domain death agonist; BIM, BCL-2–like 11; FOXO3a, forkhead box O3; JNK, c-Jun N-terminal kinase; MOA, mechanism of action; MTA, microtubule-targeting agent; P, phosphorylated; PUMA, p53-upregulated modulator of apoptosis; U, ubiquitinated.

The GR belongs to the nuclear receptor superfamily (14, 28) and is a ubiquitously expressed ligand-activated transcription factor (14, 26). In the absence of a ligand, the GR resides in the cell’s cytoplasm as a monomer, part of a large multiprotein complex that includes HSP90, immunophilins (e.g., FKBP51 and FKBP52), and other chaperone proteins (Fig. 1A; refs. 14, 26, 29). This complex maintains the GR in a conformation that has a high affinity for ligand binding. Cortisol, a lipophilic compound, freely diffuses across the cell membrane and binds a hydrophobic pocket in the ligand-binding domain (LBD) near the c-terminus of the GR protein. Binding of cortisol introduces a significant conformational change in the LBD, trapping the ligand and creating an optimal surface for coactivator protein binding, an essential change for the receptor’s transcriptional activation function (13, 14, 26, 29). The conformational change also dissociates the HSP90 chaperone complex, allowing homodimerization of the GR and exposing nuclear localization signals. The GR is then rapidly and actively transported into the nucleus through nuclear pores.

The GR homodimer binds a general consensus 15-base pair DNA sequence 5′-GGTACAnnnTGTTCT-3′ (in which nnn is a 3-base pair spacer) referred to as a glucocorticoid response element (GRE; ref. 14). To activate or suppress transcription, ligand- and DNA-bound GR forms a transcriptional complex with multiple coactivator or corepressor proteins. The nature of this transcriptional complex varies and determines the magnitude of the transcriptional response. Factors that influence the makeup of the transcriptional complex include the ligand bound to the GR, the specific GRE, accessibility within the local chromatin environment, the cellular context (cell type, differentiation state, cellular signaling activity, posttranslational modifications of the GR and its regulators, and other transcription factors), and the concentration/availability of coregulators (29, 39, 40). As such, the effect of GR activation is both ligand- and context-specific, allowing this canonical signaling pathway to have pleiotropic effects involving multiple distinct physiologic processes.

## Dysregulation of GR Signaling in Cancer

In solid tumors, dysregulated GR signaling drives prosurvival transcriptional programs that promote tumor growth, invasion, and resistance to therapy. The *NR3C1* gene encoding the GR shows little evidence for functional somatic driver mutations or epigenetic changes in human tumors. Seven percent of endometrial cancers have inactivating nonsense or missense mutations in *NR3C1*, and 6% of renal clear cell carcinomas show evidence for amplifications involving the *NR3C1* in The Cancer Genome Atlas, as analyzed using cBioPortal (RRID: SCR 014555), but the functional consequences of these changes have not been studied (41–43). Genes encoding the synthesis of endogenous GR ligands, or GR coregulators, are not the target of recurrent somatic alterations in human tumors (cBioPortal, RRID: SCR 014555). Outside of functional endocrine tumors, there is limited evidence for autocrine or paracrine signaling through the GR in the tumor microenvironment through direct synthesis by CYP11B1 or recycling from inactive metabolites by 11β-HSD1 (44–48). Yet, the concentrations of glucocorticoid produced in the tumor microenvironment are 10- to 200-fold lower than circulating cortisol and may be of limited physiologic relevance (49). Multiple splice variants of GR have been reported, as have alternate transcripts originating from different start sites, including the GRβ splice variant that lacks a ligand-binding domain (29). However, the physiologic significance of these forms of the GR is uncertain given their limited expression pattern and ligand/DNA-binding properties (50). There is no evidence for alternate splicing or transcript forms having a role in tumor initiation, promotion, or metastasis.

The GR is widely expressed in normal tissues, and its expression can be upregulated by hormones, cytokines, and chronic stress (51). Consequently, numerous tumor cell types consistently exhibit GR (52) expression, even without genetic amplification or active oncogenic signaling mechanisms. Preclinical work using synthetic glucocorticoids suggest that normal endogenous glucocorticoid levels are sufficient to activate the GR, as the morning serum cortisol range of 276 to 552 nmol/L is higher than the concentration at which half-maximal GR activation occurs (9.5 nmol/L; refs. 53–55). The GR provides prosurvival signals in malignant solid tumors (56, 57), promotes tumor growth (58) and invasion (59), drives metastasis (60), and confers resistance to chemotherapy (61–64). Consistent with these functional observations, higher tumor cell GR expression is associated with poor outcomes in patients with ovarian and endometrial cancers (65–67).

A Mendelian randomization study investigated the causal association between 3 genetic variants associated with morning plasma cortisol levels at the genome-wide significance level (*P* < 5 × 10^−8^) in the Cortisol Network consortium and the risk of various cancers in datasets from large-scale cancer consortia, the UK Biobank, and the FinnGen consortium (68). No significant associations were observed for cancer overall or breast, colorectal, lung, ovarian, or prostate cancer. The notable exception was an increased risk of endometrial cancer associated with higher genetically predicted cortisol levels [odds ratio, 1.5; 95% confidence interval (CI), 1.13–1.99; *P* = 0.005] in a meta-analysis of the endometrial cancer consortium and the FinnGen datasets (68). This association may be driven by an increase in estrogen or testosterone, known risk factors for endometrial cancer, which are associated with endogenous hypercortisolism. Alternatively, the association between plasma cortisol and endometrial cancer might be mediated by obesity, which is strongly associated with an increased risk of endometrial cancer and may be caused by elevated cortisol. Additional mediators of the observed effect might be hyperglycemia and hyperinsulinemia, which have been implicated in endometrial cancer pathogenesis and are a direct effect of cortisol dysregulation. However, the observed association did not materially change after multivariate analyses controlling genetic predictions for these factors. Further research is necessary to elucidate the potential mechanism linking hypercortisolism to endometrial cancer development.

A small proportion of patients with cancer have tumors that produce ectopic CRH, ACTH, or cortisol, causing a paraneoplastic syndrome due to extremely high levels of cortisol (and/or mineralocorticoids). With a few exceptions, such as lung carcinoids (69), these functioning endocrine tumors have consistently worse outcomes than nonfunctioning endocrine tumors of the same histology and cell of origin. Adrenal cortical cancers that secrete cortisol recur faster (relative risk 1.43-fold), and patients with these secreting adrenal cortical cancers have a shorter survival (relative risk 1.71-fold) than patients with nonsecreting adrenal cortical cancers (70). Pituitary adenomas that secrete ACTH have double the mortality risk versus nonsecretors [hazard ratio (HR) for age-adjusted mortality 2.35; ref. 71]. Ectopic ACTH secretion by other tumor types (e.g., small-cell lung, thyroid, and pancreatic cancers) is often associated with advanced, aggressive disease with short overall survival (OS), estimated at <4 months for small-cell lung cancer (SCLC) in one study (72–74). The impact may be due to immune suppression, metabolic derangements, resistance to chemotherapy, and diagnostic delay secondary to the cortisol excess. As such, hormone-secreting tumors often require more aggressive management due to their poorer prognosis, but they are rare.

Beyond patients with functional endocrine tumors, there is evidence for disruption of the HPA in patients with cancer due to physical stress, persistent pain, and debilitating fatigue and the accompanying psychologic stress from the diagnosis, fear of recurrence, associated morbidity, cost of care, and impending mortality (75, 76). Treatment-related factors also play a significant role in altering cortisol levels and GR signaling in patients with cancer. Synthetic glucocorticoids are frequently used to support cancer therapy, treating chemotherapy-induced nausea and vomiting and inflammation (77, 78). The administration of these exogenous steroids can lead to iatrogenic Cushing syndrome. Clinically, a meta-analysis of 83,614 patients with a wide range of solid cancers has shown that those who receive supportive care with exogenous glucocorticoids have a significantly shorter OS and progression-free survival (PFS; ref. 79). Possible mechanisms for these adverse outcomes include a tumor-intrinsic role for corticosteroids, the impact of corticosteroids on the immune system, or side effects of excess corticosteroids use on normal body systems.

Defining elevated cortisol in patients with cancer is a challenge due to its diurnal variation. In general, patients with cancer do not have dramatically elevated plasma cortisol levels. However, increases in cortisol, a flattening of the diurnal slope of cortisol, and/or elevated evening cortisol are reported in cohorts of patients with many different solid tumor types (80–83). Dysregulation of cortisol has been associated with feelings of hopelessness, depression, fatigue, disability (83), more advanced disease, and shorter survival in patients with many different tumor types (83, 84), including breast cancer (80), renal cell carcinoma (85), non-SCLC (NSCLC; ref. 86), and ovarian cancer (87). These are retrospective exploratory subset analyses, often using different methodologies and cut-offs to define cortisol dysregulation, making it challenging to confirm the findings or exclude publication bias. However, mechanistic animal models show evidence for a stress–cortisol–cancer axis, whereby stress increases endogenous glucocorticoid production, which in turn drives the growth and/or metastasis of cancer cells (88–94) through tumor-intrinsic and -immune mechanisms (60, 82, 95, 96). Thus, the totality of the data indicates a direct link between stress, GR signaling, and poor outcomes for patients with cancer.

## The Role of the GR in Resistance to Cytotoxic Chemotherapy

GR signaling plays a key role in chemoresistance by inducing antiapoptotic signaling pathways that enable cancer cells to evade cell death. GR signaling reduces tumor sensitivity to chemotherapy by increasing the expression of antiapoptotic proteins, such as SGK1 and DUSP1 (Fig. 1A; refs. 54, 63, 65, 94, 97, 98). Upon ligand binding, the GR translocates into the nucleus, where it binds glucocorticoid response elements and recruits specific coregulatory complexes. This interaction reshapes the local chromatin architecture and regulates the transcription of downstream target genes, including *SGK1* and *DUSP1* (Fig. 1A; ref. 33). Increased expression of SGK1 and DUSP1 inactivates the FOXO3a and JNK pathways, which are necessary for apoptosis, and modulates the activity of antiapoptotic BCL-2 family members (Fig. 1A; refs. 31–33). These molecular changes are consequences of GR activation and allow cancer cells to survive cytotoxic damage without undergoing apoptosis and induce resistance to chemotherapy. In support of this, dexamethasone treatment of GR-expressing ovarian cancer cell lines suppressed paclitaxel-induced cell death and upregulated SGK1 and DUSP1 (30). In patients with ovarian cancer, dexamethasone infusion elevated *SGK1* and *DUSP1* gene expression in tumor specimens (30).

Data derived from experimental systems with physiologic levels of glucocorticoids suggest that the role of GR signaling in developing chemotherapy resistance may apply broadly to all patients with solid tumors that express the GR. However, it is likely that the effects are exacerbated in patients with cancer with dysregulated cortisol signaling and in patients receiving exogenous glucocorticoids to support the use of anticancer therapies. Due to the widespread use of exogenous glucocorticoids in this setting, delineating the precise contribution of physiologic cortisol and exogenous glucocorticoids is challenging with the current evidence base. Two main classes of synthetic pharmacologic GR antagonists have entered clinical development: steroidal antiprogestins (mifepristone and its analogs) and selective nonsteroidal glucocorticoid receptor antagonists (“-corilants,” e.g., relacorilant and exicorilant; Supplementary Table S2). These structurally distinct drug classes have a similar potency and activity at the GR but have distinct coregulator interactomes (99) and distinct selectivity, which likely account for their differential clinical activity and therapeutic window. Among the selective nonsteroidal glucocorticoid receptor antagonists, relacorilant (CORT125134) has the most mature dataset to date; little data have been published on other compounds in this class, and generalizability across agents remains uncertain. As such, most of the discussion in this article will use relacorilant as an example.

The potent and highly selective GR antagonist, relacorilant [Ki 0.15 nmol/L for GR binding; IC_50_ 5.6–7.2 nmol/L for GR antagonism, and Ki >1,000 nmol/L for binding of androgen receptor (AR), progesterone receptor (PR), and mineralocorticoid receptor], was developed from a nonsteroidal scaffold (99, 100). Relacorilant reversibly binds the ligand-binding domain of the GR, inhibiting the effects of GR agonists but allowing dimerization of the GR, nuclear entry, and recruitment of a distinct interactome of coregulators to GR agonists or mifepristone (99). When bound to relacorilant, GR does not activate transcription at canonical GR response elements, demonstrating that relacorilant is a functional antagonist (99). Selective GR antagonists (SGRA) such as relacorilant, and less selective mifepristone analogs, such as ORIC-101, show synergy with several types of cytotoxic chemotherapy, including gemcitabine, taxanes, and platinum agents (54, 61, 101, 102). *In vitro* and *in vivo* GR inhibition suppresses SGK1 and DUSP1 expression (Fig. 1B; refs. 54, 63, 93) and reverses the chemotherapy-resistant epithelial-to-mesenchymal transition phenotype (103–105). This leads to greater chemotherapy-induced apoptosis when SGRAs are combined with chemotherapy (54) and significant tumor growth inhibition, including regression in some nonclinical models (54, 101, 105). In a phase I clinical trial, the addition of relacorilant to nab-paclitaxel in 73 patients with advanced solid tumors (75% of whom had received a prior taxane) achieved a 16% objective response rate (ORR) in all tumor types with a 4.6 months median PFS in patients with ovarian cancer (61). The duration of benefit was longer for relacorilant plus nab-paclitaxel than the prior taxane-containing regimen in 29% of patients (61). In a phase II trial (CORT125134-552), the addition of an intermittent schedule of relacorilant to nab-paclitaxel in patients with platinum-resistant ovarian cancer extended PFS (HR, 0.66; 95% CI, 0.44–0.98; *P* = 0.038) with a trend to improved OS (*P* = 0.066; ref. 37). In a recently disclosed phase III trial, ROSELLA, patients receiving relacorilant plus nab-paclitaxel had a statistically significant improvement in PFS by blinded independent central review (BICR) compared with nab-paclitaxel monotherapy (HR, 0.70; 95% CI, 0.54–0.91; *P* = 0.008; ref. 106). At an interim analysis, there was a clinically meaningful improvement in OS with the addition of relacorilant to nab-paclitaxel (HR, 0.69; 95% CI, 0.52–0.92; median 16 vs. 11.5 months; *P* = 0.012; ref. 106). At the final analysis, the combination resulted in a statistically and clinically significant improvement in OS compared with nab-paclitaxel alone (HR, 0.65; 95% CI, 0.51–0.83; median 16 vs. 11.9 months; *P* = 0.0004; ref. 107). In the phase II relacorilant trial, reductions in SGK1 and DUSP1 were observed in whole blood, providing evidence of a pharmacodynamic link to the proposed antiapoptotic mechanism (37, 108). ORIC-101 was also evaluated in combination with nab-paclitaxel in a phase I study but showed a more modest benefit in patients who had progressed on a prior taxane, with a 3.2% ORR across all tumor types and a 2.4 months median PFS in patients with ovarian cancer (101), reflecting the more modest pharmacodynamic effects seen for ORIC-101 (109) and the reduced selectivity when compared with relacorilant (ORIC-101 has an IC_50_ of 21 nmol/L for the PR whereas relacorilant has no affinity at 1,000 nmol/L; refs. 100, 109).

These nonclinical, translational, and clinical observations support a role for SGRAs in sensitizing tumors to chemotherapy and overcoming chemotherapy resistance in ovarian cancer. Similar nonclinical data have been reported in triple-negative breast cancer (56, 58, 64, 94, 110), and patients with SCLC brain metastases are reported to have worse outcomes to teniposide with corticosteroid use (111), suggesting that these observations may apply more broadly to advanced solid tumors. In addition to chemoresistance being mediated through SGK1 and DUSP1 in triple-negative breast cancer (93, 94), the GR may also act through the YAP oncogene to promote survival and reduce sensitivity to cytotoxic agents (112). The reported synergistic effects of PARP inhibitors with mifepristone in patient-derived xenografts also suggest that SGRAs may have further potential in maintenance therapy for patients with ovarian cancer (113).

## GR Signaling and Resistance to AR Inhibition

The AR is the lineage-defining transcription factor for the luminal prostate epithelium (114, 115). The GR and AR are both nuclear hormone receptors with near-identical consensus DNA-binding sites. They regulate the transcriptional activity of an overlapping group of genes (116, 117). Androgen signaling is required for the initiation and progression of early prostate cancer (118). Chemical castration (GnRH modulators), antiandrogens (abiraterone), and AR inhibitors (enzalutamide and apalutamide) are mainstays of prostate cancer therapy (119). Eventually, though, resistance to antiandrogen therapy develops through multiple mechanisms, including mutations that reduce the affinity of AR inhibitors, ligand-independent AR signaling (120), and parallel oncogenic signaling pathways (116).

The AR directly represses GR expression (121), and GR expression increases in prostate cancers following exposure to AR inhibitors in cell lines, human tumor xenograft models, and in the clinic (116). Therefore, there is a strong biological rationale that GR activity allows prostate cancers to bypass their dependence on AR signaling and thereby develop resistance to AR inhibitors. Activation of GR reduces the efficacy of AR inhibitors and accelerates the development of resistance, whereas GR antagonism has the opposite effect in nonclinical models (116). Nevertheless, this is a complex context-dependent biology; corticosteroids can also have modest anticancer activity in combination with chemotherapy (122, 123) and in patients progressing on abiraterone (124), secondary to lower adrenal androgen production, resulting in stabilization or modest reductions in prostate-specific antigen (PSA) levels (125). In other contexts, GR agonists may directly activate mutant forms of the AR (such as L701H) that can arise secondary to androgen deprivation therapy (126).

Inhibition of the GR has been evaluated in clinical trials of patients with prostate cancer. Initially, the nonselective GR antagonist mifepristone was used. Mifepristone antagonizes GR in the presence of physiologic ligand concentrations, with an IC_50_ value of 3.3 nmol/L in a GR luciferase antagonist assay (109), but can also show context-dependent partial agonism, with a maximal effect of up to 25% of dexamethasone (127). Mifepristone has off-target activity against several other nuclear hormone receptors, with antagonism (IC_50_ 7.8 nmol/L) of the AR (128). In the absence of androgens, high concentrations of mifepristone (100 nmol/L) have weak partial agonist activity against the AR (15%–20% of the response of the potent agonist R1881 at 0.01 nmol/L; ref. 129), but in other contexts, mifepristone has no agonistic activity (128), and functional assays suggest minimal agonist activity at therapeutically relevant concentrations (130). Monotherapy treatment with mifepristone in 19 men with castration-resistant prostate cancer showed no clinical activity, but a significant increase in circulating androgens (testosterone and dihydrotestosterone increased by 80%–90%) was observed secondary to a negative feedback loop driving increased ACTH production (131). When mifepristone was combined with enzalutamide versus enzalutamide alone in a randomized phase II trial of 66 men with metastatic castration-resistant prostate cancer, no difference in the rate of radiographic or PSA progression was observed (132). Like the monotherapy mifepristone study, a 2.5-fold increase in circulating testosterone was observed in patients that received mifepristone, with the potential to drive tumor growth and adversely affect efficacy (132). Mifepristone analogs, such as ORIC-101 (109), have also failed to show meaningful anticancer activity in prostate cancer (133). When ORIC-101 was combined with enzalutamide in 41 patients with metastatic castration-resistant prostate cancer who had progressed on enzalutamide, no meaningful antitumor effect was observed (133).

In healthy subjects, relacorilant was able to completely suppress increases in circulating FKBP5 and GILZ secondary to a physiologic dose of prednisone (134). In a phase Ib/II study, relacorilant was combined with enzalutamide in heavily pretreated patients with metastatic castration-resistant prostate cancer that had progressed following treatment with an antiandrogen (135). Thirty-five patients were enrolled; 94% had received prior abiraterone, 89% at least one AR inhibitor, 9% both enzalutamide and apalutamide, 69% docetaxel, and 49% cabazitaxel. All patients remained castrate, with no increases in testosterone levels observed on treatment. Twelve patients were evaluable for a PSA response at 12 weeks, of whom 4 (33%) achieved at least a 50% reduction in PSA. Twenty-two patients were evaluable by RECIST, of whom 13 (59%) had the best radiographic response of stable disease; there were no objective responses. The median PSA-defined PFS was short at 2.7 months; radiographic median PFS was 1.8 months. However, 4 patients stayed on treatment for >6 months, with time on therapy of 11, 17.5, 22.1 (ongoing), and 38.1 (ongoing) months at the time of the primary analysis, respectively. These data are not sufficient to support further development of relacorilant in an unselected population of patients with heavily pretreated metastatic castration-resistant prostate cancer. However, the data suggest that combined inhibition of the AR and GR may have a role in a selected group of patients and/or in a less treatment-refractory setting.

Building on these initial findings, a randomized phase II trial evaluating the addition of relacorilant to enzalutamide compared with enzalutamide alone was initiated in patients with high-risk localized prostate cancer (NCT05726292). Patients received 6 months of therapy prior to surgical resection with the aim of improving pathologic response and reducing recurrence rates with the addition of relacorilant. The nature of the study facilitates the collection of tissue and blood biomarkers before and after treatment to help identify predictive biomarkers of clinical benefit. If positive, these results may inform the design of a pivotal phase III study in patients with advanced prostate cancer.

## GR Signaling in Estrogen-Driven Tumors

The GR shares structural homology, chromatin binding sites, and coregulatory networks with the estrogen receptor (ER). Consequently, the functional impact of GR signaling in estrogen-responsive malignancies, particularly breast and endometrial tumors, is dependent on ER expression and activity. In endometrial cancer, expression of the GR is associated with a worse prognosis but only in tumors that express high levels of the ER (136). More than one-third of DNA binding by the GR in endometrial cancer cells is altered by ER activation, suggesting a complex transcriptional regulatory interplay between the receptors (136).

Interactions between activated GR and ER, with cross-receptor influences on transcriptional programs, have also been reported in breast cancer (137–139). In patients with ER+ breast cancers, high GR expression and a signature of GR activation are both associated with improved outcomes (97, 140), unlike triple-negative breast cancers in which high GR expression is a poor prognostic factor (141). GR agonists can reduce circulating estradiol in mice bearing MCF7 xenografts by increasing the expression of estrogen sulfotransferase in tumor cells and the liver (142). Moreover, the GR can tether and displace the ER from estrogen DNA response elements in the promoters of canonical target genes such as *CCND1*, leading to reduced expression of proliferative genes (139, 143). The GR can also reduce the expression of wild-type and mutant *ESR1*, reducing the proliferation and metastasis of *ESR1*-mutant breast cancer xenografts (140). Finally, the GR can modulate the ER transcriptional program, leading to an increase in ER-driven transcription of tumor suppressors such as *ZBTB16* (144). These findings were recently confirmed by Padrão and colleagues (145), who also showed that GR activation can enhance the efficacy of tamoxifen in ER+ breast cancer models. Intriguingly ligand binding of the GR by synthetic GR modulators with either antagonist or partial agonist activity can also suppress tumor growth, replicating the effects of GR liganded by a glucocorticoid agonist in ER+ breast cancer models (146). However, in PI3K-mutant PTEN-null breast cancer cells, dexamethasone reduces tumor growth and mifepristone rescues this effect (147). In contrast to luminal A breast cancers, luminal B tumors do not seem to be sensitive to GR activity (144). The role of the GR in lobular breast cancers seems to mirror ER+ ductal carcinomas, yet GR activation results in an enhanced metastatic phenotype while slowing the tumor growth rate. GR overexpression in SUM44 xenografts suppresses primary mammary tumor growth and paradoxically increases bone metastasis (148). Together, these data show that the directionality of GR modulation is highly context-dependent in ER+ breast cancer; therapeutic development should proceed cautiously with a clear understanding of tumor biology to ensure an appropriate benefit–risk profile.

## GR Signaling in Immuno-oncology

Immune surveillance is an important mechanism to control tumor growth and metastasis through coordinated innate and adaptive immune responses (149). However, tumors frequently develop mechanisms to evade or suppress antitumor immunity. Glucocorticoids suppress immune responses (51), including T-cell proliferation and function, and interferon gamma (IFNγ) and tumor necrosis factor alfa (TNFα) release (150–154). Increased glucocorticoid levels alter the cytokine milieu from a proinflammatory to an anti-inflammatory profile, leading to the reprogramming of protective type 1 T-helper (Th1) cells toward anti-inflammatory type 2 T-helper (Th2) cells and immunosuppressive regulatory T cells (Treg; refs. 154–156). These effects of endogenous and synthetic glucocorticoids interfere with immune surveillance and the elimination of cells containing non–self-antigens, including cancer cells (27, 51).

**Figure 2.**
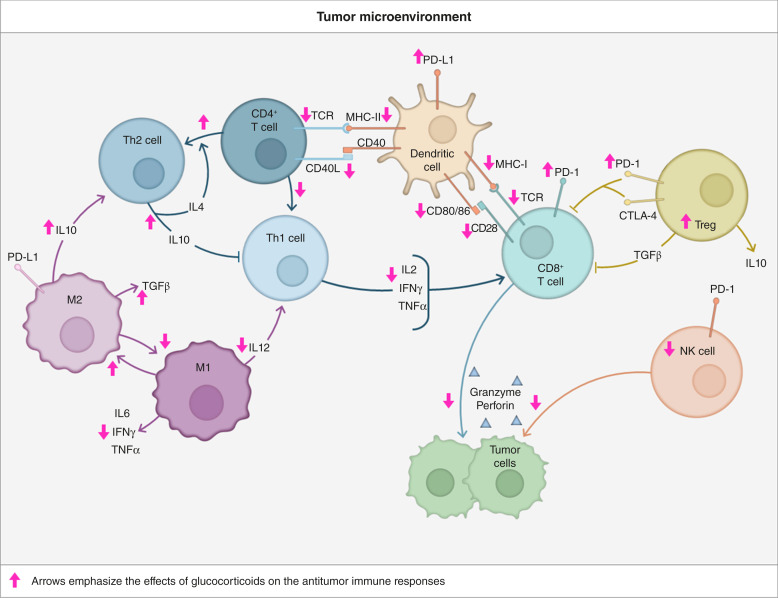
Effects of glucocorticoids on antitumor immune responses. TCR-mediated activation of immune responses forms a crucial aspect of antitumor immunity. DCs are antigen-presenting cells that contribute to the activation of CD4^+^ and CD8^+^ T cells through interactions between MHC class molecules and TCR and costimulatory molecules CD28, CD80/86, CD40, and CD40L. Glucocorticoids inhibit DC maturation and costimulation of T cells via decreased expression of MHC classes I and II, CD28 and CD80/86 molecules, interference with TCR signaling, and reduced CD40-mediated stimulatory potential (51, 154, 166). DCs stimulate Th cell antitumor responses, whereas Treg cells have a protumor effect via release of IL10 and TGFβ. Glucocorticoids prevent Th1 differentiation and direct the T-cell response toward a Th2 phenotype (potentially through downregulation of IL12 and upregulation of IL4 and IL10 cytokines) and promote Treg activity (51, 154, 166). Glucocorticoids suppress T-cell function by inhibiting the production of proinflammatory cytokines such as IL2, TNFα, and IFNγ (166). Activated CD8^+^ T cells and NK cells contribute to tumor cell death via granzymes and perforin; glucocorticoids inhibit both pathways (166, 167). M1 macrophages are responsible for tumor cell clearance, whereas M2 macrophages exert a tumor-promoting effect. Glucocorticoids inhibit M1 polarization of macrophages and promote switching to the M2 phenotype, thereby decreasing proinflammatory cytokines (e.g., IL6, IL12, and TNFα) and increasing anti-inflammatory cytokines (e.g., IL10 and TGFβ; ref. 166). Immune checkpoint proteins like PD-1 and its ligand PD-L1 play a key role in evading antitumor immune mechanisms, and drugs inhibiting this interaction have approved indications for many tumor types (157–161). Glucocorticoids may induce PD-1 and PD-L1 expression, thereby inhibiting cytotoxic T-cell activation (166). CTLA-4, cytotoxic T cell–associated protein-4; DC, dendritic cell; MHC, major histocompatibility complex; NK, natural killer; TCR, T-cell receptor; TGFβ, transforming growth factor β; Th1/2, T helper 1/2.

Immune checkpoint inhibitors target key immune regulators that tumors use to evade immunosurveillance and have approved indications for many tumor types (Fig. 2; refs. 157–161). Programmed cell death protein 1 (PD-1) is an immune checkpoint that binds to its ligands, PD-1 ligand 1 (PD-L1) and PD-1 ligand 2 (PD-L2), and thereby suppresses T cell–mediated antitumor responses, increasing cancer growth and metastases (162). Although blockade of PD-1 signaling has transformed the care of many patients, a majority of patients have a suboptimal response or no clinical benefit (163–165).

In syngeneic tumors grown in immunocompetent mice, dexamethasone accelerates tumor growth (168), and in an autochthonous pancreatic cancer mouse model, corticosterone (the murine equivalent of cortisol) abolishes immunotherapy responses (169). In a prospective observational clinical study, dysregulated endogenous cortisol was reported to blunt the response to anti–PD(L)-1 therapy (86). Higher peak doses of glucocorticoids used to manage emergent immune-related adverse events are associated with worse outcomes in patients with a range of solid tumor indications (170). In addition, the use of glucocorticoids prior to the start of immune checkpoint inhibitor therapy or early in the first cycle is associated with worse outcomes in NSCLC (171–173).

In tumor cells, inhibition of GR increases expression of MHC-I, enhancing tumor antigen presentation and leading to increased infiltration of CD8^+^ T cells (174, 175). Thus, targeting the GR has the potential to turn “cold” (immune-desert) tumors “hot” (immune-inflamed), extending the efficacy of anticancer immunotherapies to additional tumor types that have shown suboptimal benefit to date. The selective knockout of *NR3C1* in T-effector cells in both hot and cold syngeneic models leads to an increase in effector function, increased tumor growth inhibition, loss of T-cell exhaustion markers (LAG3, TIM3, TIGIT, and PD-1) but no change in T-cell infiltration (45). These data show that GR antagonism has the potential to reactivate T cells in tumors following the failure of immune checkpoint therapy. GR deletion in FOXP3+ Tregs reduces the suppressive phenotype of tumor-infiltrating Treg cells and leads to tumor growth inhibition in syngeneic models (46). Although not yet demonstrated in the context of solid tumors, GR expression is increased on myeloid-derived suppressor cells in experimental models of immune tolerance. Dexamethasone increases and mifepristone decreases the suppressive activity of myeloid-derived suppressor cells in mixed lymphocyte reactions (176). Dexamethasone also suppresses PD-L1 expression by neutrophils, monocytes, and dendritic cells in the tumor microenvironment of breast cancer allograft models (145). Moreover, in animal models, tumor-associated monocytes and macrophages have been shown to express CYP11A1 or 11β-HSD1, supporting their role as a source of glucocorticoids in the tumor microenvironment (45, 48). Thus, GR antagonism may also have utility in tumors that have a suppressive immune milieu.

If agonism of the GR reduces the efficacy of anticancer immunotherapy, it is rational to consider that selective antagonism of the GR may enhance the efficacy of anti–PD(L)-1 antibodies. Stress-elevated endogenous glucocorticoids blocked IFN responses in antigen-presenting cells and IFNγ T-cell activation in nonclinical models (82). Administration of a GR antagonist reversed the adverse therapeutic impact of stress and restored therapeutic control. In nonclinical *ex vivo* experiments, SGRAs reversed the immunosuppressive effects of corticosteroids on proinflammatory cytokine release in human peripheral blood mononuclear cells (53). The combination of an SGRA and anti–PD-1 reduced tumor growth compared with the anti–PD-1 antibody alone in a mouse syngeneic tumor model (53). SGRA effects were associated with increased cytotoxic CD8^+^ T-cell responses against tumor antigens and elevated expression of proinflammatory cytokines such as TNFα, concomitant with decreased levels of anti-inflammatory cytokines, such as interleukin 10 (IL10). In patients with cancer, GR antagonism decreases circulating IL8 (also known as C-X-C motif chemokine ligand 8), a neutrophil chemotactic factor that exerts direct protumorigenic effects, including the promotion of angiogenesis, epithelial-to-mesenchymal transition, and invasion and/or metastasis (177, 178). In prostate cancer models, dexamethasone is associated with an increase in infiltrating monocytes/macrophages that express IL8 (179). This effect can be reversed with mifepristone treatment. GR antagonism also decreases the circulating neutrophil-to-lymphocyte ratio (*Data on file*, Corcept Therapeutics Inc.; refs. 53, 95). Lower IL8 and a lower neutrophil-to-lymphocyte ratio are both associated with improved outcomes in patients treated with anti–PD(L)-1 therapy (180). Neutrophil-mediated changes in the tumor microenvironment secondary to chronic stress and increased cortisol signaling also enhance tumor metastasis in nonclinical models, reinforcing a direct link between IL8, neutrophil biology, cancer immunotherapy, and GR activation (95).

These data suggest that GR antagonism can reverse the immunosuppressive and protumorigenic effects of glucocorticoids and potentiate anticancer responses to anti–PD(L)-1 immunotherapies. However, glucocorticoids are a mainstay of treatment for patients with immune-related adverse events, which occur in approximately half of all patients receiving an anti–PD(L)-1 agent, with ∼15% of patients requiring high-dose corticosteroid treatment (181). Therefore, close monitoring will be required when evaluating such combinations to ensure an appropriate benefit–risk profile.

## Considerations for the Use of Glucocorticoids in Supportive Care

To date, no randomized controlled trial has assessed the effect of supportive glucocorticoid use on survival outcomes in patients with solid tumors. Maintaining >80% to 85% of planned dose intensity has been associated with improved outcomes in patients receiving nonadjuvant chemotherapy regimens (182). This indirect evidence supports the use of glucocorticoids to manage treatment-emergent toxicities, avoiding dose reductions and interruptions. However, prolonged or repeated use carries an increased risk of diabetes, dyslipidemia, hypertension, cardiac disease, adrenal insufficiency on withdrawal, muscle atrophy, central obesity, infection risk, autoimmune hematologic conditions, gastrointestinal bleeding, osteoporosis, and neuropsychiatric conditions (183). In addition, iatrogenic glucocorticoids are associated with reduced survival in certain cancer treatment settings (reviewed above). Therefore, wherever possible, steroid use should be minimized. Given their directly opposing mechanisms of action, concurrent administration of glucocorticoids and SGRAs should be avoided. Often glucocorticoids are introduced early in the clinical development of new drugs to manage hypersensitivity reactions as part of a cocktail of supportive care, but the need for steroids is rarely revisited. Steroid sparing chemotherapy, such as nab-paclitaxel, avoids these issues, and it is often possible to safely omit dexamethasone with repeat administration of drugs that are known to cause hypersensitivity (184). Given the benefit–risk implications, the use of glucocorticoids urgently requires further study in randomized controlled trials and appropriate updates to clinical guidelines.

## Limitations

The findings presented here are derived largely from preclinical evidence and may not fully reflect the complexity of human tumor biology. There is limited clinical data on SGRAs, especially data supporting mechanisms other than the apoptotic effects described herein, and data on compounds other than relacorilant, mifepristone, and ORIC-101. More prospective clinical studies are needed to confirm their effects across tumor types and under different treatment contexts.

## Future Implications and Conclusions

The impact of the human stress response on outcomes for patients with cancer has been known for more than 35 years (3), yet a mechanistic understanding of why this occurs and clinical data to support the therapeutic potential of this pathway have been wanting. The development of SGRAs (54, 100) has deepened our translational and clinical understanding of resistance to AR inhibition (135), chemotherapy (37), and targeted therapy (113), yielding therapeutic breakthroughs (106). Furthermore, improvements in OS and PFS for an SGRA in several randomized controlled trials (37, 106) in patients with advanced ovarian cancer have focused attention on the potential to improve patient outcomes more broadly. A greater appreciation for the potential of immunotherapy and the impact of glucocorticoids on the efficacy of immune checkpoint inhibitors (53, 82, 86, 95, 170, 172, 173, 185) begs further exploration.

GR coregulators have a complex and diverse role, which has a differential impact on GR signaling depending on the cellular context. Recent studies in glioblastoma models have shown that this may be even more complex, with some tumors directly co-opting the body’s circadian cortisol rhythm to drive growth (186). Predicting the exact tissues and processes that will be affected by a SGRA is therefore challenging but could yield new mechanistic insights and therapeutic opportunities (187). The potential of this fundamental physiologic pathway is deserving of more study to refine and develop mechanistically differentiated SGRAs for future clinical research.

## Supplementary Material

Supplemental Table 1 Opposing roles of the glucocorticoid receptor pathway in lymphoid cancers versus solid tumors and its clinical implications

Supplemental Table 2 Glucocorticoid receptor antagonists assessed in patients with various solid tumors

## References

[1] Williams K , Jackson SE, Beeken RJ, Steptoe A, Wardle J. The impact of a cancer diagnosis on health and well-being: a prospective, population-based study. Psychooncology 2016;25:626–32. 26425927 10.1002/pon.3998 PMC4915489

[2] Xia W , Ou M, Chen Y, Chen F, Yan M, Xiao Z, . Experiences of patients with advanced cancer coping with chronic pain: a qualitative analysis. BMC Palliat Care 2024;23:94. 38600476 10.1186/s12904-024-01418-2 PMC11005139

[3] Spiegel D , Bloom JR, Kraemer HC, Gottheil E. Effect of psychosocial treatment on survival of patients with metastatic breast cancer. Lancet 1989;2:888–91. 2571815 10.1016/s0140-6736(89)91551-1

[4] Stagl JM , Lechner SC, Carver CS, Bouchard LC, Gudenkauf LM, Jutagir DR, . A randomized controlled trial of cognitive-behavioral stress management in breast cancer: survival and recurrence at 11-year follow-up. Breast Cancer Res Treat 2015;154:319–28. 26518021 10.1007/s10549-015-3626-6 PMC5752103

[5] Andersen BL , Yang HC, Farrar WB, Golden-Kreutz DM, Emery CF, Thornton LM, . Psychologic intervention improves survival for breast cancer patients: a randomized clinical trial. Cancer 2008;113:3450–8. 19016270 10.1002/cncr.23969 PMC2661422

[6] Fu WW , Popovic M, Agarwal A, Milakovic M, Fu TS, McDonald R, . The impact of psychosocial intervention on survival in cancer: a meta-analysis. Ann Palliat Med 2016;5:93–106. 27121737 10.21037/apm.2016.03.06

[7] Smith TB , Workman C, Andrews C, Barton B, Cook M, Layton R, . Effects of psychosocial support interventions on survival in inpatient and outpatient healthcare settings: a meta-analysis of 106 randomized controlled trials. PLoS Med 2021;18:e1003595. 34003832 10.1371/journal.pmed.1003595 PMC8130925

[8] Iversen HG , Hjort G. The influence of corticoid steroids on the frequency of spleen metastases in patients with breast cancer. Acta Pathol Microbiol Scand 1958;44:205–12. 13594480 10.1111/j.1699-0463.1958.tb01070.x

[9] Sherlock P , Hartmann WH. Adrenal steroids and the pattern of metastases of breast cancer. JAMA 1962;181:313–7. 13911707 10.1001/jama.1962.03050300033007

[10] Choi J , Ceribelli M, Phelan JD, Haupl B, Huang DW, Wright GW, . Molecular targets of glucocorticoids that elucidate their therapeutic efficacy in aggressive lymphomas. Cancer Cell 2024;42:833–49.e12. 38701792 10.1016/j.ccell.2024.04.007 PMC11168741

[11] Burger JA , Montserrat E. Coming full circle: 70 years of chronic lymphocytic leukemia cell redistribution, from glucocorticoids to inhibitors of B-cell receptor signaling. Blood 2013;121:1501–9. 23264597 10.1182/blood-2012-08-452607 PMC4968370

[12] Inaba H , Pui CH. Glucocorticoid use in acute lymphoblastic leukaemia. Lancet Oncol 2010;11:1096–106. 20947430 10.1016/S1470-2045(10)70114-5 PMC3309707

[13] Kadmiel M , Cidlowski JA. Glucocorticoid receptor signaling in health and disease. Trends Pharmacol Sci 2013;34:518–30. 23953592 10.1016/j.tips.2013.07.003 PMC3951203

[14] Timmermans S , Souffriau J, Libert C. A general introduction to glucocorticoid biology. Front Immunol 2019;10:1545. 31333672 10.3389/fimmu.2019.01545 PMC6621919

[15] Zefferino R , Di Gioia S, Conese M. Molecular links between endocrine, nervous and immune system during chronic stress. Brain Behav 2021;11:e01960. 33295155 10.1002/brb3.1960 PMC7882157

[16] Herman JP , McKlveen JM, Ghosal S, Kopp B, Wulsin A, Makinson R, . Regulation of the hypothalamic-pituitary-adrenocortical stress response. Compr Physiol 2016;6:603–21. 27065163 10.1002/cphy.c150015 PMC4867107

[17] Girotti M , Adler SM, Bulin SE, Fucich EA, Paredes D, Morilak DA. Prefrontal cortex executive processes affected by stress in health and disease. Prog Neuropsychopharmacol Biol Psychiatry 2018;85:161–79. 28690203 10.1016/j.pnpbp.2017.07.004 PMC5756532

[18] Schwabe L , Höffken O, Tegenthoff M, Wolf OT. Stress-induced enhancement of response inhibition depends on mineralocorticoid receptor activation. Psychoneuroendocrinology 2013;38:2319–26. 23831264 10.1016/j.psyneuen.2013.05.001

[19] Rahdar A , Galván A. The cognitive and neurobiological effects of daily stress in adolescents. Neuroimage 2014;92:267–73. 24553284 10.1016/j.neuroimage.2014.02.007

[20] Mbiydzenyuy NE , Qulu LA. Stress, hypothalamic-pituitary-adrenal axis, hypothalamic-pituitary-gonadal axis, and aggression. Metab Brain Dis 2024;39:1613–36. 39083184 10.1007/s11011-024-01393-w PMC11535056

[21] Knezevic E , Nenic K, Milanovic V, Knezevic NN. The role of cortisol in chronic stress, neurodegenerative diseases, and psychological disorders. Cells 2023;12:2726. 38067154 10.3390/cells12232726 PMC10706127

[22] Ramamoorthy S , Cidlowski JA. Corticosteroids: mechanisms of action in health and disease. Rheum Dis Clin North Am 2016;42:15–31, vii. 26611548 10.1016/j.rdc.2015.08.002 PMC4662771

[23] Dziurkowska E , Wesolowski M. Cortisol as a biomarker of mental disorder severity. J Clin Med 2021;10:5204. 34768724 10.3390/jcm10215204 PMC8584322

[24] Lockett J , Inder WJ, Clifton VL. The glucocorticoid receptor: isoforms, functions, and contribution to glucocorticoid sensitivity. Endocr Rev 2024;45:593–624. 38551091 10.1210/endrev/bnae008 PMC11244253

[25] Pivonello R , Isidori AM, De Martino MC, Newell-Price J, Biller BM, Colao A. Complications of Cushing’s syndrome: state of the art. Lancet Diabetes Endocrinol 2016;4:611–29. 27177728 10.1016/S2213-8587(16)00086-3

[26] Clarisse D , Van Moortel L, Van Leene C, Gevaert K, De Bosscher K. Glucocorticoid receptor signaling: intricacies and therapeutic opportunities. Trends Biochem Sci 2024;49:431–44. 38429217 10.1016/j.tibs.2024.01.012

[27] Khadka S , Druffner SR, Duncan BC, Busada JT. Glucocorticoid regulation of cancer development and progression. Front Endocrinol (Lausanne) 2023;14:1161768. 37143725 10.3389/fendo.2023.1161768 PMC10151568

[28] Fadel L , Dacic M, Fonda V, Sokolsky BA, Quagliarini F, Rogatsky I, . Modulating glucocorticoid receptor actions in physiology and pathology: insights from coregulators. Pharmacol Ther 2023;251:108531. 37717739 10.1016/j.pharmthera.2023.108531 PMC10841922

[29] Oakley RH , Cidlowski JA. The biology of the glucocorticoid receptor: new signaling mechanisms in health and disease. J Allergy Clin Immunol 2013;132:1033–44. 24084075 10.1016/j.jaci.2013.09.007 PMC4084612

[30] Melhem A , Yamada SD, Fleming GF, Delgado B, Brickley DR, Wu W, . Administration of glucocorticoids to ovarian cancer patients is associated with expression of the anti-apoptotic genes SGK1 and MKP1/DUSP1 in ovarian tissues. Clin Cancer Res 2009;15:3196–204. 19383827 10.1158/1078-0432.CCR-08-2131 PMC4707040

[31] Dhanasekaran DN , Reddy EP. JNK signaling in apoptosis. Oncogene 2008;27:6245–51. 18931691 10.1038/onc.2008.301 PMC3063296

[32] Liu Y , Ao X, Ding W, Ponnusamy M, Wu W, Hao X, . Critical role of FOXO3a in carcinogenesis. Mol Cancer 2018;17:104. 30045773 10.1186/s12943-018-0856-3 PMC6060507

[33] Buonaiuto R , Neola G, Cecere SC, Caltavituro A, Cefaliello A, Pietroluongo E, . Glucocorticoid receptor and ovarian cancer: from biology to therapeutic intervention. Biomolecules 2023;13:653. 37189400 10.3390/biom13040653 PMC10135974

[34] Jordan MA , Wilson L. Microtubules as a target for anticancer drugs. Nat Rev Cancer 2004;4:253–65. 15057285 10.1038/nrc1317

[35] Kaur R , Kaur G, Gill RK, Soni R, Bariwal J. Recent developments in tubulin polymerization inhibitors: an overview. Eur J Med Chem 2014;87:89–124. 25240869 10.1016/j.ejmech.2014.09.051

[36] Suleimenov M , Bekbayev S, Ten M, Suleimenova N, Tlegenova M, Nurmagambetova A, . Bcl-xL activity influences outcome of the mitotic arrest. Front Pharmacol 2022;13:933112. 36188556 10.3389/fphar.2022.933112 PMC9520339

[37] Colombo N , Van Gorp T, Matulonis UA, Oaknin A, Grisham RN, Fleming GF, . Relacorilant + nab-paclitaxel in patients with recurrent, platinum-resistant ovarian cancer: a three-arm, randomized, controlled, open-label phase II study. J Clin Oncol 2023;41:4779–89. 37364223 10.1200/JCO.22.02624 PMC10602497

[38] Prekovic S , Zwart W. Inhibiting the glucocorticoid receptor to enhance chemotherapy response. J Clin Oncol 2023;41:4790–3. 37535891 10.1200/JCO.23.01195

[39] Quatrini L , Ugolini S. New insights into the cell- and tissue-specificity of glucocorticoid actions. Cell Mol Immunol 2021;18:269–78. 32868909 10.1038/s41423-020-00526-2 PMC7456664

[40] Stallcup MR , Poulard C. Gene-specific actions of transcriptional coregulators facilitate physiological plasticity: evidence for a physiological coregulator code. Trends Biochem Sci 2020;45:497–510. 32413325 10.1016/j.tibs.2020.02.006 PMC7230073

[41] Cerami E , Gao J, Dogrusoz U, Gross BE, Sumer SO, Aksoy BA, . The cBio cancer genomics portal: an open platform for exploring multidimensional cancer genomics data. Cancer Discov 2012;2:401–4. 22588877 10.1158/2159-8290.CD-12-0095 PMC3956037

[42] de Bruijn I , Kundra R, Mastrogiacomo B, Tran TN, Sikina L, Mazor T, . Analysis and visualization of longitudinal genomic and clinical data from the AACR Project GENIE Biopharma Collaborative in cBioPortal. Cancer Res 2023;83:3861–7. 37668528 10.1158/0008-5472.CAN-23-0816 PMC10690089

[43] Gao J , Aksoy BA, Dogrusoz U, Dresdner G, Gross B, Sumer SO, . Integrative analysis of complex cancer genomics and clinical profiles using the cBioPortal. Sci Signal 2013;6:pl1. 23550210 10.1126/scisignal.2004088 PMC4160307

[44] Sidler D , Renzulli P, Schnoz C, Berger B, Schneider-Jakob S, Flück C, . Colon cancer cells produce immunoregulatory glucocorticoids. Oncogene 2011;30:2411–9. 21258413 10.1038/onc.2010.629

[45] Acharya N , Madi A, Zhang H, Klapholz M, Escobar G, Dulberg S, . Endogenous glucocorticoid signaling regulates CD8^+^ T cell differentiation and development of dysfunction in the tumor microenvironment. Immunity 2020;53:658–71.e6. 32937153 10.1016/j.immuni.2020.08.005 PMC7682805

[46] Taves MD , Otsuka S, Taylor MA, Donahue KM, Meyer TJ, Cam MC, . Tumors produce glucocorticoids by metabolite recycling, not synthesis, and activate Tregs to promote growth. J Clin Invest 2023;133:e164599. 37471141 10.1172/JCI164599 PMC10503810

[47] Drebert Z , De Vlieghere E, Bridelance J, De Wever O, De Bosscher K, Bracke M, . Glucocorticoids indirectly decrease colon cancer cell proliferation and invasion via effects on cancer-associated fibroblasts. Exp Cell Res 2018;362:332–42. 29196164 10.1016/j.yexcr.2017.11.034

[48] Poinot H , Dupuychaffray E, Arnoux G, Alvarez M, Tachet J, Ezzar O, . Activation of endogenous glucocorticoids by HSD11B1 inhibits the antitumor immune response in renal cancer. Oncoimmunology 2024;13:2286820. 38170044 10.1080/2162402X.2023.2286820 PMC10761155

[49] Weitzman ED , Fukushima D, Nogeire C, Roffwarg H, Gallagher TF, Hellman L. Twenty-four hour pattern of the episodic secretion of cortisol in normal subjects. J Clin Endocrinol Metab 1971;33:14–22. 4326799 10.1210/jcem-33-1-14

[50] de Castro M , Elliot S, Kino T, Bamberger C, Karl M, Webster E, . The non-ligand binding beta-isoform of the human glucocorticoid receptor (hGR beta): tissue levels, mechanism of action, and potential physiologic role. Mol Med 1996;2:597–607. 8898375 PMC2230188

[51] Cain DW , Cidlowski JA. Immune regulation by glucocorticoids. Nat Rev Immunol 2017;17:233–47. 28192415 10.1038/nri.2017.1 PMC9761406

[52] Block TS , Murphy TI, Munster PN, Nguyen DP, Lynch FJ. Glucocorticoid receptor expression in 20 solid tumor types using immunohistochemistry assay. Cancer Manag Res 2017;9:65–72. 28293120 10.2147/CMAR.S124475 PMC5345989

[53] Greenstein AE , Hunt HJ. The glucocorticoid receptor modulator relacorilant reverses the immunosuppressive effects of cortisol. Int Immunopharmacol 2023;120:110312. 37230031 10.1016/j.intimp.2023.110312

[54] Greenstein AE , Hunt HJ. Glucocorticoid receptor antagonism promotes apoptosis in solid tumor cells. Oncotarget 2021;12:1243–55. 34194622 10.18632/oncotarget.27989 PMC8238250

[55] Mulatero P , Panarelli M, Schiavone D, Rossi A, Mengozzi G, Kenyon CJ, . Impaired cortisol binding to glucocorticoid receptors in hypertensive patients. Hypertension 1997;30:1274–8. 9369287 10.1161/01.hyp.30.5.1274

[56] Moran TJ , Gray S, Mikosz CA, Conzen SD. The glucocorticoid receptor mediates a survival signal in human mammary epithelial cells. Cancer Res 2000;60:867–72. 10706096

[57] Wu W , Pew T, Zou M, Pang D, Conzen SD. Glucocorticoid receptor-induced MAPK phosphatase-1 (MPK-1) expression inhibits paclitaxel-associated MAPK activation and contributes to breast cancer cell survival. J Biol Chem 2005;280:4117–24. 15590693 10.1074/jbc.M411200200

[58] Conway ME , McDaniel JM, Graham JM, Guillen KP, Oliver PG, Parker SL, . STAT3 and GR cooperate to drive gene expression and growth of basal-like triple-negative breast cancer. Cancer Res 2020;80:4355–70. 32816914 10.1158/0008-5472.CAN-20-1379 PMC9462839

[59] Sood AK , Bhatty R, Kamat AA, Landen CN, Han L, Thaker PH, . Stress hormone–mediated invasion of ovarian cancer cells. Clin Cancer Res 2006;12:369–75. 16428474 10.1158/1078-0432.CCR-05-1698 PMC3141061

[60] Obradović MMS , Hamelin B, Manevski N, Couto JP, Sethi A, Coissieux M-M, . Glucocorticoids promote breast cancer metastasis. Nature 2019;567:540–4. 30867597 10.1038/s41586-019-1019-4

[61] Munster PN , Greenstein AE, Fleming GF, Borazanci E, Sharma MR, Custodio JM, . Overcoming taxane resistance: preclinical and phase 1 studies of relacorilant, a selective glucocorticoid receptor modulator, with nab-paclitaxel in solid tumors. Clin Cancer Res 2022;28:3214–24. 35583817 10.1158/1078-0432.CCR-21-4363 PMC9662918

[62] Reeder A , Attar M, Nazario L, Bathula C, Zhang A, Hochbaum D, . Stress hormones reduce the efficacy of paclitaxel in triple negative breast cancer through induction of DNA damage. Br J Cancer 2015;112:1461–70. 25880007 10.1038/bjc.2015.133 PMC4453678

[63] Stringer-Reasor EM , Baker GM, Skor MN, Kocherginsky M, Lengyel E, Fleming GF, . Glucocorticoid receptor activation inhibits chemotherapy-induced cell death in high-grade serous ovarian carcinoma. Gynecol Oncol 2015;138:656–62. 26115975 10.1016/j.ygyno.2015.06.033 PMC4556542

[64] West DC , Kocherginsky M, Tonsing-Carter EY, Dolcen DN, Hosfield DJ, Lastra RR, . Discovery of a glucocorticoid receptor (GR) activity signature using selective GR antagonism in ER-negative breast cancer. Clin Cancer Res 2018;24:3433–46. 29636357 10.1158/1078-0432.CCR-17-2793 PMC6530562

[65] Veneris JT , Darcy KM, Mhawech-Fauceglia P, Tian C, Lengyel E, Lastra RR, . High glucocorticoid receptor expression predicts short progression-free survival in ovarian cancer. Gynecol Oncol 2017;146:153–60. 28456378 10.1016/j.ygyno.2017.04.012 PMC5955699

[66] Tangen IL , Veneris JT, Halle MK, Werner HM, Trovik J, Akslen LA, . Expression of glucocorticoid receptor is associated with aggressive primary endometrial cancer and increases from primary to metastatic lesions. Gynecol Oncol 2017;147:672–7. 28927900 10.1016/j.ygyno.2017.09.013

[67] Veneris JT , Huang L, Churpek JE, Conzen SD, Fleming GF. Glucocorticoid receptor expression is associated with inferior overall survival independent of BRCA mutation status in ovarian cancer. Int J Gynecol Cancer 2019;29:357–64. 30683758 10.1136/ijgc-2018-000101

[68] Larsson SC , Lee WH, Kar S, Burgess S, Allara E. Assessing the role of cortisol in cancer: a wide-ranged Mendelian randomisation study. Br J Cancer 2021;125:1025–9. 34316022 10.1038/s41416-021-01505-8 PMC8476513

[69] La Rosa S , Volante M, Uccella S, Maragliano R, Rapa I, Rotolo N, . ACTH-producing tumorlets and carcinoids of the lung: clinico-pathologic study of 63 cases and review of the literature. Virchows Arch 2019;475:587–97. 31264037 10.1007/s00428-019-02612-x

[70] Vanbrabant T , Fassnacht M, Assie G, Dekkers OM. Influence of hormonal functional status on survival in adrenocortical carcinoma: systematic review and meta-analysis. Eur J Endocrinol 2018;179:429–36. 30325179 10.1530/EJE-18-0450

[71] Dekkers OM , Biermasz NR, Pereira AM, Roelfsema F, van Aken MO, Voormolen JH, . Mortality in patients treated for Cushing’s disease is increased, compared with patients treated for nonfunctioning pituitary macroadenoma. J Clin Endocrinol Metab 2007;92:976–81. 17200171 10.1210/jc.2006-2112

[72] Al-Toubah T , Pelle E, Hallanger-Johnson J, Haider M, Strosberg J. ACTH-secreting pancreatic neuroendocrine neoplasms: a case-series. J Neuroendocrinol 2023;35:e13336. 37688510 10.1111/jne.13336

[73] Koehler VF , Fuss CT, Berr CM, Frank-Raue K, Raue F, Hoster E, . Medullary thyroid cancer with ectopic Cushing’s syndrome: a multicentre case series. Clin Endocrinol (Oxf) 2022;96:847–56. 34743368 10.1111/cen.14617

[74] Shepherd FA , Laskey J, Evans WK, Goss PE, Johansen E, Khamsi F. Cushing’s syndrome associated with ectopic corticotropin production and small-cell lung cancer. J Clin Oncol 1992;10:21–7. 1309381 10.1200/JCO.1992.10.1.21

[75] Armer JS , Clevenger L, Davis LZ, Cuneo M, Thaker PH, Goodheart MJ, . Life stress as a risk factor for sustained anxiety and cortisol dysregulation during the first year of survivorship in ovarian cancer. Cancer 2018;124:3401–8. 29905941 10.1002/cncr.31570 PMC6108904

[76] Cuneo MG , Schrepf A, Slavich GM, Thaker PH, Goodheart M, Bender D, . Diurnal cortisol rhythms, fatigue and psychosocial factors in five-year survivors of ovarian cancer. Psychoneuroendocrinology 2017;84:139–42. 28711723 10.1016/j.psyneuen.2017.06.019 PMC5575993

[77] Djedovic V , Lee YY, Kollara A, May T, Brown TJ. The two faces of adjuvant glucocorticoid treatment in ovarian cancer. Horm Cancer 2018;9:95–107. 29313170 10.1007/s12672-017-0319-0 PMC10355875

[78] Kim KN , LaRiviere M, Macduffie E, White CA, Jordan-Luft MM, Anderson E, . Use of glucocorticoids in patients with cancer: potential benefits, harms, and practical considerations for clinical practice. Pract Radiat Oncol 2023;13:28–40. 35917896 10.1016/j.prro.2022.07.003

[79] Petrelli F , Bukovec R, Perego G, Luisa R, Luciani A, Zaniboni A, . Association of steroid use with survival in solid tumours. Eur J Cancer 2020;141:105–14. 33130548 10.1016/j.ejca.2020.09.020

[80] Sephton SE , Sapolsky RM, Kraemer HC, Spiegel D. Diurnal cortisol rhythm as a predictor of breast cancer survival. J Natl Cancer Inst 2000;92:994–1000. 10861311 10.1093/jnci/92.12.994

[81] van der Pompe G , Antoni MH, Heijnen CJ. Elevated basal cortisol levels and attenuated ACTH and cortisol responses to a behavioral challenge in women with metastatic breast cancer. Psychoneuroendocrinology 1996;21:361–74. 8844875 10.1016/0306-4530(96)00009-1

[82] Yang H , Xia L, Chen J, Zhang S, Martin V, Li Q, . Stress-glucocorticoid-TSC22D3 axis compromises therapy-induced antitumor immunity. Nat Med 2019;25:1428–41. 31501614 10.1038/s41591-019-0566-4

[83] Weinrib AZ , Sephton SE, Degeest K, Penedo F, Bender D, Zimmerman B, . Diurnal cortisol dysregulation, functional disability, and depression in women with ovarian cancer. Cancer 2010;116:4410–9. 20564155 10.1002/cncr.25299 PMC3118555

[84] Bakour N , Moriarty F, Moore G, Robson T, Annett SL. Prognostic significance of glucocorticoid receptor expression in cancer: a systematic review and meta-analysis. Cancers (Basel) 2021;13:1649. 33916028 10.3390/cancers13071649 PMC8037088

[85] Rasmuson T , Ljungberg B, Grankvist K, Jacobsen J, Olsson T. Increased serum cortisol levels are associated with high tumour grade in patients with renal cell carcinoma. Acta Oncol 2001;40:83–7. 11321667 10.1080/028418601750071118

[86] Zeng Y , Hu CH, Li YZ, Zhou JS, Wang SX, Liu MD, . Association between pretreatment emotional distress and immune checkpoint inhibitor response in non-small-cell lung cancer. Nat Med 2024;30:1680–8. 38740994 10.1038/s41591-024-02929-4 PMC11186781

[87] Schrepf A , Thaker PH, Goodheart MJ, Bender D, Slavich GM, Dahmoush L, . Diurnal cortisol and survival in epithelial ovarian cancer. Psychoneuroendocrinology 2015;53:256–67. 25647344 10.1016/j.psyneuen.2015.01.010 PMC4440672

[88] Agosin M , Christen R, Badinez O, Gasic G, Neghme A, Pizarro O, . Cortisone-induced metastases of adenocarcinoma in mice. Proc Soc Exp Biol Med 1952;80:128–31. 14930094 10.3181/00379727-80-19544

[89] Baserga R , Shubik P. The action of cortisone on transplanted and induced tumors in mice. Cancer Res 1954;14:12–6. 13126926

[90] Baserga R , Shubik P. Action of cortisone on disseminated tumor cells after removal of the primary growth. Science 1955;121:100–1. 13225747 10.1126/science.121.3134.100

[91] Pomeroy TC . Studies on the mechanism of cortisone-induced metastases of transplantable mouse tumors. Cancer Res 1954;14:201–4. 13150334

[92] Seshadri M , Poduval T, Sundaram K. Studies on metastases. I. Role of sensitization and immunosuppression. J Natl Cancer Inst 1979;63:1205–10. 291748

[93] Pang D , Kocherginsky M, Krausz T, Kim SY, Conzen SD. Dexamethasone decreases xenograft response to Paclitaxel through inhibition of tumor cell apoptosis. Cancer Biol Ther 2006;5:933–40. 16775428 10.4161/cbt.5.8.2875

[94] Skor MN , Wonder EL, Kocherginsky M, Goyal A, Hall BA, Cai Y, . Glucocorticoid receptor antagonism as a novel therapy for triple-negative breast cancer. Clin Cancer Res 2013;19:6163–72. 24016618 10.1158/1078-0432.CCR-12-3826 PMC3860283

[95] He X-Y , Gao Y, Ng D, Michalopoulou E, George S, Adrover JM, . Chronic stress increases metastasis via neutrophil-mediated changes to the microenvironment. Cancer Cell 2024;42:474–86.e12. 38402610 10.1016/j.ccell.2024.01.013 PMC11300849

[96] Powell CB , Mutch DG, Kao MS, Kao JW, Perry DL, Westphale E, . Dexamethasone used as an antiemetic in chemotherapy protocols inhibits natural cytotoxic (NC) cell activity. Cancer 1990;65:466–72. 2297637 10.1002/1097-0142(19900201)65:3<466::aid-cncr2820650315>3.0.co;2-q

[97] Pan D , Kocherginsky M, Conzen SD. Activation of the glucocorticoid receptor is associated with poor prognosis in estrogen receptor-negative breast cancer. Cancer Res 2011;71:6360–70. 21868756 10.1158/0008-5472.CAN-11-0362 PMC3514452

[98] Zhang C , Wenger T, Mattern J, Ilea S, Frey C, Gutwein P, . Clinical and mechanistic aspects of glucocorticoid-induced chemotherapy resistance in the majority of solid tumors. Cancer Biol Ther 2007;6:278–87. 17224649 10.4161/cbt.6.2.3652

[99] Viho EMG , Kroon J, Feelders RA, Houtman R, van den Dungen ESR, Pereira AM, . Peripheral glucocorticoid receptor antagonism by relacorilant with modest HPA axis disinhibition. J Endocrinol 2023;256:e220263. 36445262 10.1530/JOE-22-0263 PMC9874980

[100] Hunt HJ , Belanoff JK, Walters I, Gourdet B, Thomas J, Barton N, . Identification of the clinical candidate (R)-(1-(4-Fluorophenyl)-6-((1-methyl-1H-pyrazol-4-yl)sulfonyl)-4,4a,5,6,7,8-hexahydro-1H-pyrazolo[3,4-g]isoquinolin-4a-yl)(4-(trifluoromethyl)pyridin-2-yl)methanone (CORT125134): a selective glucocorticoid receptor (GR) antagonist. J Med Chem 2017;60:3405–21. 28368581 10.1021/acs.jmedchem.7b00162

[101] Chen CT , Khanna V, Kummar S, Abdul-Karim RM, Sommerhalder D, Tolcher AW, . ORIC-101, a glucocorticoid receptor antagonist, in combination with nab-paclitaxel in patients with advanced solid tumors. Cancer Res Commun 2024;4:2415–26. 39177285 10.1158/2767-9764.CRC-24-0115 PMC11396014

[102] Zhou H , Ye Q, Pankov A, Kong W, Barkund S, Friedman LS, . ORIC-101 robustly inhibits the glucocorticoid pathway and overcomes chemoresistance in triple-negative breast cancer. Cancer Res 2020;80 (4_Supplement):P6-03-24.

[103] Cortes J , Schöffski P, Littlefield BA. Multiple modes of action of eribulin mesylate: emerging data and clinical implications. Cancer Treat Rev 2018;70:190–8. 30243063 10.1016/j.ctrv.2018.08.008

[104] Dongre A , Weinberg RA. New insights into the mechanisms of epithelial-mesenchymal transition and implications for cancer. Nat Rev Mol Cell Biol 2019;20:69–84. 30459476 10.1038/s41580-018-0080-4

[105] Zhou H , Barkund S, Pankov A, Hegde G, Kong W, Narayanan P, . Abstract 4121: ORIC-101 overcomes resistance to diverse chemotherapeutics across cancer types. Cancer Res 2020;80(16_Supplement):4121.

[106] Olawaiye AB , Gladieff L, O’Malley DM, Kim JW, Garbaos G, Salutari V, . Relacorilant and nab-paclitaxel in patients with platinum-resistant ovarian cancer (ROSELLA): an open-label, randomised, controlled, phase 3 trial. Lancet 2025;405:2205–16. 40473448 10.1016/S0140-6736(25)01040-2

[107] Lorusso D , Gladieff L, O’Malley DM, Kim J-W, Garbaos G, Fagotti A, . Overall survival with relacorilant and nab-paclitaxel in patients with platinum-resistant ovarian cancer (ROSELLA): a phase 3 randomised controlled trial. Lancet 2026;407(10538):1513–24. 41974149 10.1016/S0140-6736(26)00462-9

[108] Lorusso D , Greenstein A, Wadekar S, Tudor I, Hunt H, Guyer B. 524MO glucocorticoid receptor expression and activity in a phase II ovarian cancer trial of the glucocorticoid receptor modulator relacorilant in combination with nab-paclitaxel. Ann Oncol 2022;33:S784–5.

[109] Rew Y , Du X, Eksterowicz J, Zhou H, Jahchan N, Zhu L, . Discovery of a potent and selective steroidal glucocorticoid receptor antagonist (ORIC-101). J Med Chem 2018;61:7767–84. 30091920 10.1021/acs.jmedchem.8b00743

[110] He L , Yuan L, Sun Y, Wang P, Zhang H, Feng X, . Glucocorticoid receptor signaling activates TEAD4 to promote breast cancer progression. Cancer Res 2019;79:4399–411. 31289134 10.1158/0008-5472.CAN-19-0012

[111] Postmus PE , Smit EF, Haaxma-Reiche H, van Zandwijk N, Ardizzoni A, Quoix E, . Teniposide for brain metastases of small-cell lung cancer: a phase II study. European Organization for Research and Treatment of Cancer Lung Cancer Cooperative Group. J Clin Oncol 1995;13:660–5. 7884426 10.1200/JCO.1995.13.3.660

[112] Sorrentino G , Ruggeri N, Zannini A, Ingallina E, Bertolio R, Marotta C, . Glucocorticoid receptor signalling activates YAP in breast cancer. Nat Commun 2017;8:14073. 28102225 10.1038/ncomms14073 PMC5253666

[113] Zhang X , Yao J, Li X, Niu N, Liu Y, Hajek RA, . Targeting polyploid giant cancer cells potentiates a therapeutic response and overcomes resistance to PARP inhibitors in ovarian cancer. Sci Adv 2023;9:eadf7195. 37478190 10.1126/sciadv.adf7195 PMC10361597

[114] Wang Q , Li W, Zhang Y, Yuan X, Xu K, Yu J, . Androgen receptor regulates a distinct transcription program in androgen-independent prostate cancer. Cell 2009;138:245–56. 19632176 10.1016/j.cell.2009.04.056 PMC2726827

[115] Vickman RE , Franco OE, Moline DC, Vander Griend DJ, Thumbikat P, Hayward SW. The role of the androgen receptor in prostate development and benign prostatic hyperplasia: a review. Asian J Urol 2020;7:191–202. 32742923 10.1016/j.ajur.2019.10.003 PMC7385520

[116] Arora VK , Schenkein E, Murali R, Subudhi SK, Wongvipat J, Balbas MD, . Glucocorticoid receptor confers resistance to antiandrogens by bypassing androgen receptor blockade. Cell 2013;155:1309–22. 24315100 10.1016/j.cell.2013.11.012 PMC3932525

[117] Sahu B , Laakso M, Pihlajamaa P, Ovaska K, Sinielnikov I, Hautaniemi S, . FoxA1 specifies unique androgen and glucocorticoid receptor binding events in prostate cancer cells. Cancer Res 2013;73:1570–80. 23269278 10.1158/0008-5472.CAN-12-2350

[118] Gao J , Arnold JT, Isaacs JT. Conversion from a paracrine to an autocrine mechanism of androgen-stimulated growth during malignant transformation of prostatic epithelial cells. Cancer Res 2001;61:5038–44. 11431338

[119] Horwich A , Hugosson J, de Reijke T, Wiegel T, Fizazi K, Kataja V, . Prostate cancer: ESMO Consensus Conference Guidelines 2012. Ann Oncol 2013;24:1141–62. 23303340 10.1093/annonc/mds624

[120] Herberts C , Annala M, Sipola J, Ng SW, Chen XE, Nurminen A, . Deep whole-genome ctDNA chronology of treatment-resistant prostate cancer. Nature 2022;608:199–208. 35859180 10.1038/s41586-022-04975-9

[121] Xie N , Cheng H, Lin D, Liu L, Yang O, Jia L, . The expression of glucocorticoid receptor is negatively regulated by active androgen receptor signaling in prostate tumors. Int J Cancer 2015;136:E27–38. 25138562 10.1002/ijc.29147

[122] Tannock IF , Osoba D, Stockler MR, Ernst DS, Neville AJ, Moore MJ, . Chemotherapy with mitoxantrone plus prednisone or prednisone alone for symptomatic hormone-resistant prostate cancer: a Canadian randomized trial with palliative end points. J Clin Oncol 1996;14:1756–64. 8656243 10.1200/JCO.1996.14.6.1756

[123] Kantoff PW , Halabi S, Conaway M, Picus J, Kirshner J, Hars V, . Hydrocortisone with or without mitoxantrone in men with hormone-refractory prostate cancer: results of the cancer and leukemia group B 9182 study. J Clin Oncol 1999;17:2506. 10561316 10.1200/JCO.1999.17.8.2506

[124] Romero-Laorden N , Lozano R, Jayaram A, López-Campos F, Saez MI, Montesa A, . Phase II pilot study of the prednisone to dexamethasone switch in metastatic castration-resistant prostate cancer (mCRPC) patients with limited progression on abiraterone plus prednisone (SWITCH study). Br J Cancer 2018;119:1052–9. 30131546 10.1038/s41416-018-0123-9 PMC6219494

[125] Khandwala HM , Vassilopoulou-Sellin R, Logethetis CJ, Friend KE. Corticosteroid-induced inhibition of adrenal androgen production in selected patients with prostate cancer. Endocr Pract 2001;7:11–5. 11250762 10.4158/EP.7.1.11

[126] Zhao X-Y , Malloy PJ, Krishnan AV, Swami S, Navone NM, Peehl DM, . Glucocorticoids can promote androgen-independent growth of prostate cancer cells through a mutated androgen receptor. Nat Med 2000;6:703–6. 10835690 10.1038/76287

[127] Zhang S , Jonklaas J, Danielsen M. The glucocorticoid agonist activities of mifepristone (RU486) and progesterone are dependent on glucocorticoid receptor levels but not on EC50 values. Steroids 2007;72:600–8. 17509631 10.1016/j.steroids.2007.03.012

[128] Muddana SS , Price AM, MacBride MM, Peterson BR. 11β-Alkyl-Δ9-19-nortestosterone derivatives: high-Affinity ligands and potent partial agonists of the androgen receptor. J Med Chem 2004;47:4985–8. 15456242 10.1021/jm0342515

[129] Song LN , Coghlan M, Gelmann EP. Antiandrogen effects of mifepristone on coactivator and corepressor interactions with the androgen receptor. Mol Endocrinol 2004;18:70–85. 14593076 10.1210/me.2003-0189

[130] Safi R , Wardell SE, Watkinson P, Qin X, Lee M, Park S, . Androgen receptor monomers and dimers regulate opposing biological processes in prostate cancer cells. Nat Commun 2024;15:7675. 39227594 10.1038/s41467-024-52032-y PMC11371910

[131] Taplin ME , Manola J, Oh WK, Kantoff PW, Bubley GJ, Smith M, . A phase II study of mifepristone (RU-486) in castration-resistant prostate cancer, with a correlative assessment of androgen-related hormones. BJU Int 2008;101:1084–9. 18399827 10.1111/j.1464-410X.2008.07509.x

[132] Serritella AV , Shevrin D, Heath EI, Wade JL, Martinez E, Anderson A, . Phase I/II trial of enzalutamide and mifepristone, a glucocorticoid receptor antagonist, for metastatic castration-resistant prostate cancer. Clin Cancer Res 2022;28:1549–59. 35110415 10.1158/1078-0432.CCR-21-4049 PMC9012680

[133] Abida W , Hahn AW, Shore N, Agarwal N, Sieber P, Smith MR, . Phase I study of ORIC-101, a glucocorticoid receptor antagonist, in combination with enzalutamide in patients with metastatic castration-resistant prostate cancer progressing on enzalutamide. Clin Cancer Res 2024;30:1111–20. 38226958 10.1158/1078-0432.CCR-23-3508 PMC10947849

[134] Hunt H , Donaldson K, Strem M, Zann V, Leung P, Sweet S, . Assessment of safety, tolerability, pharmacokinetics, and pharmacological effect of orally administered CORT125134: an adaptive, double-blind, randomized, placebo-controlled phase 1 clinical study. Clin Pharmacol Drug Dev 2018;7:408–21. 28967708 10.1002/cpdd.389 PMC5947602

[135] Desai KB , Serritella AV, Stadler WM, O’Donnell PH, Sweis RF, Szmulewitz RZ. A phase I trial of enzalutamide plus selective glucocorticoid receptor modulator relacorilant in patients with metastatic castration-resistant prostate cancer. Clin Cancer Res 2024;30:2384–92. 38536082 10.1158/1078-0432.CCR-23-3636 PMC11147727

[136] Vahrenkamp JM , Yang C-H, Rodriguez AC, Almomen A, Berrett KC, Trujillo AN, . Clinical and genomic crosstalk between glucocorticoid receptor and estrogen receptor α in endometrial cancer. Cell Rep 2018;22:2995–3005. 29539426 10.1016/j.celrep.2018.02.076 PMC5870123

[137] Bolt MJ , Stossi F, Newberg JY, Orjalo A, Johansson HE, Mancini MA. Coactivators enable glucocorticoid receptor recruitment to fine-tune estrogen receptor transcriptional responses. Nucleic Acids Res 2013;41:4036–48. 23444138 10.1093/nar/gkt100 PMC3627592

[138] Miranda TB , Voss TC, Sung M-H, Baek S, John S, Hawkins M, . Reprogramming the chromatin landscape: interplay of the estrogen and glucocorticoid receptors at the genomic level. Cancer Res 2013;73:5130–9. 23803465 10.1158/0008-5472.CAN-13-0742 PMC3799864

[139] West DC , Pan D, Tonsing-Carter EY, Hernandez KM, Pierce CF, Styke SC, . GR and ER coactivation alters the expression of differentiation genes and associates with improved ER+ breast cancer outcome. Mol Cancer Res 2016;14:707–19. 27141101 10.1158/1541-7786.MCR-15-0433 PMC5008962

[140] Manivannan M , Jehanno C, Kloc M, Gomez Miragaya J, Diepenbruck M, Volkmann K, . Activated glucocorticoid receptor is an estrogen receptor silencer in ER+ metastatic breast cancer. EMBO Mol Med 2026;18:151–86. 41261233 10.1038/s44321-025-00342-z PMC12808765

[141] Chen Z , Lan X, Wu D, Sunkel B, Ye Z, Huang J, . Ligand-dependent genomic function of glucocorticoid receptor in triple-negative breast cancer. Nat Commun 2015;6:8323. 26374485 10.1038/ncomms9323 PMC4573460

[142] Gong H , Jarzynka MJ, Cole TJ, Lee JH, Wada T, Zhang B, . Glucocorticoids antagonize estrogens by glucocorticoid receptor–mediated activation of estrogen sulfotransferase. Cancer Res 2008;68:7386–93. 18794126 10.1158/0008-5472.CAN-08-1545 PMC6551207

[143] Karmakar S , Jin Y, Nagaich AK. Interaction of glucocorticoid receptor (GR) with estrogen receptor (ER) α and activator protein 1 (AP1) in dexamethasone-mediated interference of ERα activity. J Biol Chem 2013;288:24020–34. 23814048 10.1074/jbc.M113.473819 PMC3745347

[144] Prekovic S , Chalkiadakis T, Roest M, Roden D, Lutz C, Schuurman K, . Luminal breast cancer identity is determined by loss of glucocorticoid receptor activity. EMBO Mol Med 2023;15:e17737. 37902007 10.15252/emmm.202317737 PMC10701603

[145] Padrão N , Severson TM, Gregoricchio S, Guijarro A, Lutz C, Taranto D, . Fasting boosts breast cancer therapy efficacy via glucocorticoid activation. Nature 2026;649:1013–21. 41372410 10.1038/s41586-025-09869-0 PMC12823405

[146] Tonsing-Carter E , Hernandez KM, Kim CR, Harkless RV, Oh A, Bowie KR, . Glucocorticoid receptor modulation decreases ER-positive breast cancer cell proliferation and suppresses wild-type and mutant ER chromatin association. Breast Cancer Res 2019;21:82. 31340854 10.1186/s13058-019-1164-6 PMC6651939

[147] Yip HYK , Chee A, Ang CS, Shin SY, Ooms LM, Mohammadi Z, . Control of glucocorticoid receptor levels by PTEN establishes a failsafe mechanism for tumor suppression. Mol Cell 2020;80:279–95.e8. 33065020 10.1016/j.molcel.2020.09.027

[148] Porter BA , Frerich C, Lainé M, Clark AB, Durdana I, Lee J, . Glucocorticoid receptor activation in lobular breast cancer is associated with reduced cell proliferation and promotion of metastases. Cancers (Basel) 2023;15:4679. 37835373 10.3390/cancers15194679 PMC10571671

[149] Disis ML . Immune regulation of cancer. J Clin Oncol 2010;28:4531–8. 20516428 10.1200/JCO.2009.27.2146 PMC3041789

[150] Hu X , Li W-P, Meng C, Ivashkiv LB. Inhibition of IFN-γ signaling by glucocorticoids. J Immunol 2003;170:4833–9. 12707366 10.4049/jimmunol.170.9.4833

[151] Wu C-Y , Wang K, McDyer JF, Seder RA. Prostaglandin E2 and dexamethasone inhibit IL-12 receptor expression and IL-12 responsiveness. J Immunol 1998;161:2723–30. 9743329

[152] Pandolfi J , Baz P, Fernández P, Discianni Lupi A, Payaslián F, Billordo LA, . Regulatory and effector T-cells are differentially modulated by dexamethasone. Clin Immunol 2013;149:400–10. 24211714 10.1016/j.clim.2013.09.008

[153] Winiski A , Wang S, Schwendinger B, Stuetz A. Inhibition of T-cell activation in vitro in human peripheral blood mononuclear cells by pimecrolimus and glucocorticosteroids and combinations thereof. Exp Dermatol 2007;16:699–704. 17620098 10.1111/j.1600-0625.2007.00588.x

[154] Kalfeist L , Galland L, Ledys F, Ghiringhelli F, Limagne E, Ladoire S. Impact of glucocorticoid use in oncology in the immunotherapy era. Cells 2022;11:770. 35269392 10.3390/cells11050770 PMC8909189

[155] Blotta MH , DeKruyff RH, Umetsu DT. Corticosteroids inhibit IL-12 production in human monocytes and enhance their capacity to induce IL-4 synthesis in CD4^+^ lymphocytes. J Immunol 1997;158:5589–95. 9190905

[156] Elenkov IJ , Chrousos GP. Stress system-organization, physiology and immunoregulation. Neuroimmunomodulation 2006;13:257–67. 17709947 10.1159/000104853

[157] Bylicki O , Paleiron N, Margery J, Guisier F, Vergnenegre A, Robinet G, . Targeting the PD-1/PD-L1 immune checkpoint in EGFR-mutated or ALK-translocated non-small-cell lung cancer. Target Oncol 2017;12:563–9. 28624922 10.1007/s11523-017-0510-9

[158] Chikuma S . Basics of PD-1 in self-tolerance, infection, and cancer immunity. Int J Clin Oncol 2016;21:448–55. 26864303 10.1007/s10147-016-0958-0

[159] Janakiram M , Pareek V, Cheng H, Narasimhulu DM, Zang X. Immune checkpoint blockade in human cancer therapy: lung cancer and hematologic malignancies. Immunotherapy 2016;8:809–19. 27349980 10.2217/imt-2016-0001 PMC5619054

[160] Naidoo J , Page DB, Wolchok JD. Immune checkpoint blockade. Hematol Oncol Clin North Am 2014;28:585–600. 24880949 10.1016/j.hoc.2014.02.002

[161] Wang DH , Guo L, Wu XH. Checkpoint inhibitors in immunotherapy of ovarian cancer. Tumour Biol 2015;36:33–9. 25409618 10.1007/s13277-014-2848-2

[162] Alsaab HO , Sau S, Alzhrani R, Tatiparti K, Bhise K, Kashaw SK, . PD-1 and PD-L1 checkpoint signaling inhibition for cancer immunotherapy: mechanism, combinations, and clinical outcome. Front Pharmacol 2017;8:561. 28878676 10.3389/fphar.2017.00561 PMC5572324

[163] KEYTRUDA® (Pembrolizumab) . US prescribing information. Rahway (NJ): Merck & Co., Inc.; 2026.

[164] Cui JW , Li Y, Yang Y, Yang HK, Dong JM, Xiao ZH, . Tumor immunotherapy resistance: revealing the mechanism of PD-1/PD-L1-mediated tumor immune escape. Biomed Pharmacother 2024;171:116203. 38280330 10.1016/j.biopha.2024.116203

[165] Hiltbrunner S , Cords L, Kasser S, Freiberger SN, Kreutzer S, Toussaint NC, . Acquired resistance to anti-PD1 therapy in patients with NSCLC associates with immunosuppressive T cell phenotype. Nat Commun 2023;14:5154. 37620318 10.1038/s41467-023-40745-5 PMC10449840

[166] Schwarzlmueller P , Triebig A, Assié G, Jouinot A, Theurich S, Maier T, . Steroid hormones as modulators of anti-tumoural immunity. Nat Rev Endocrinol 2025;21:331–43. 40128599 10.1038/s41574-025-01102-2

[167] Krukowski K , Eddy J, Kosik KL, Konley T, Janusek LW, Mathews HL. Glucocorticoid dysregulation of natural killer cell function through epigenetic modification. Brain Behav Immun 2011;25:239–49. 20656012 10.1016/j.bbi.2010.07.244 PMC2989339

[168] Gillette RW , Wunderlich DA. Accelerated growth of mammary tumor cells in normal and athymic mice after treatment in vitro with dexamethasone. Cancer Res 1978;38:3146–9. 80260

[169] Flint TR , Janowitz T, Connell CM, Roberts EW, Denton AE, Coll AP, . Tumor-induced IL-6 reprograms host metabolism to suppress anti-tumor immunity. Cell Metab 2016;24:672–84. 27829137 10.1016/j.cmet.2016.10.010 PMC5106372

[170] Verheijden RJ , de Groot JS, Fabriek BO, Hew MN, May AM, Suijkerbuijk KP. Corticosteroids for immune-related adverse events and checkpoint inhibitor efficacy: analysis of six clinical trials. J Clin Oncol 2024;42:3713–24. 39110922 10.1200/JCO.24.00191

[171] Scott SC , Pennell NA. Early use of systemic corticosteroids in patients with advanced NSCLC treated with nivolumab. J Thorac Oncol 2018;13:1771–5. 29935305 10.1016/j.jtho.2018.06.004

[172] Arbour KC , Mezquita L, Long N, Rizvi H, Auclin E, Ni A, . Impact of baseline steroids on efficacy of programmed cell death-1 and programmed death-ligand 1 blockade in patients with non-small-cell lung cancer. J Clin Oncol 2018;36:2872–8. 30125216 10.1200/JCO.2018.79.0006

[173] Fucà G , Galli G, Poggi M, Lo Russo G, Proto C, Imbimbo M, . Modulation of peripheral blood immune cells by early use of steroids and its association with clinical outcomes in patients with metastatic non-small cell lung cancer treated with immune checkpoint inhibitors. ESMO Open 2019;4:e000457. 30964126 10.1136/esmoopen-2018-000457 PMC6435242

[174] Deng Y , Xia X, Zhao Y, Zhao Z, Martinez C, Yin W, . Glucocorticoid receptor regulates PD-L1 and MHC-I in pancreatic cancer cells to promote immune evasion and immunotherapy resistance. Nat Commun 2021;12:7041. 34873175 10.1038/s41467-021-27349-7 PMC8649069

[175] Taya M , Gray E, Srivastava T, Chee W-Y, Fleming GF, DeVilbiss AW, . Ovarian cancer cell glucocorticoid receptor activation increases myeloid-derived suppressor cell tumor infiltration. Endocrinology 2026;167(4):bqag019. 41732082 10.1210/endocr/bqag019

[176] Lu Y , Liu H, Bi Y, Yang H, Li Y, Wang J, . Glucocorticoid receptor promotes the function of myeloid-derived suppressor cells by suppressing HIF1α-dependent glycolysis. Cell Mol Immunol 2018;15:618–29. 28287112 10.1038/cmi.2017.5 PMC6079089

[177] Liu Q , Li A, Tian Y, Wu JD, Liu Y, Li T, . The CXCL8-CXCR1/2 pathways in cancer. Cytokine Growth Factor Rev 2016;31:61–71. 27578214 10.1016/j.cytogfr.2016.08.002 PMC6142815

[178] Borazanci EH , Bahary N, Chung V, Huyck TK, Kio EA, Chiorean EG, . Relacorilant plus nab-paclitaxel for the treatment of metastatic pancreatic ductal adenocarcinoma: results from the open-label RELIANT study. Oncologist 2024;29:957–65. 39191530 10.1093/oncolo/oyae210 PMC11546724

[179] Eigentler A , Handle F, Schanung S, Degen A, Hackl H, Erb HHH, . Glucocorticoid treatment influences prostate cancer cell growth and the tumor microenvironment via altered glucocorticoid receptor signaling in prostate fibroblasts. Oncogene 2024;43:235–47. 38017134 10.1038/s41388-023-02901-5 PMC10798901

[180] Schalper KA , Carleton M, Zhou M, Chen T, Feng Y, Huang SP, . Elevated serum interleukin-8 is associated with enhanced intratumor neutrophils and reduced clinical benefit of immune-checkpoint inhibitors. Nat Med 2020;26:688–92. 32405062 10.1038/s41591-020-0856-x PMC8127102

[181] Novák Z , Gold M, Herrstedt J, Gill S, Savarese A, Diaz J, . PR038/#248 management of immune-related adverse events in patients with primary advanced or recurrent endometrial cancer: dostarlimab plus chemotherapy compared with chemotherapy alone in the ENGOT-EN6-NSGO/GOG-3031/RUBY trial. Int J Gynecol Cancer 2023;33:A50–1.

[182] Nielson CM , Bylsma LC, Fryzek JP, Saad HA, Crawford J. Relative dose intensity of chemotherapy and survival in patients with advanced stage solid tumor cancer: a systematic review and meta-analysis. Oncologist 2021;26:e1609–18. 33973301 10.1002/onco.13822 PMC8417866

[183] Faggiano A , Mazzilli R, Natalicchio A, Adinolfi V, Argentiero A, Danesi R, . Corticosteroids in oncology: use, overuse, indications, contraindications. An Italian Association of Medical Oncology (AIOM)/Italian Association of Medical Diabetologists (AMD)/Italian Society of Endocrinology (SIE)/Italian Society of Pharmacology (SIF) multidisciplinary consensus position paper. Crit Rev Oncol Hematol 2022;180:103826. 36191821 10.1016/j.critrevonc.2022.103826

[184] de Castro Baccarin AL , Irene MN, de Iracema Gomes Cubero D, Luz AS, Castro SN, Sordi R, . The feasibility of dexamethasone omission in weekly paclitaxel treatment for breast cancer patients. Support Care Cancer 2019;27:927–31. 30069696 10.1007/s00520-018-4381-0

[185] He XY , Ng D, Van Aelst L, Egeblad M. Stressing out about cancer immunotherapy. Cancer Cell 2019;36:468–70. 31715130 10.1016/j.ccell.2019.10.013

[186] Gonzalez-Aponte MF , Damato AR, Simon T, Aripova N, Darby F, Jeon MS, . Daily glucocorticoids promote glioblastoma growth and circadian synchrony to the host. Cancer Cell 2025;43:144–60.e7. 39672168 10.1016/j.ccell.2024.11.012 PMC11732716

[187] Meijer OC , Koorneef LL, Kroon J. Glucocorticoid receptor modulators. Ann Endocrinol (Paris) 2018;79:107–11. 29731108 10.1016/j.ando.2018.03.004

