# Supplementary material for: Signaling Under Stress: Targeting the Glucocorticoid Receptor in Cancer
Source: Cancer Res. 2026 May 18;86(15):3636–48. doi: 10.1158/0008-5472.CAN-25-4426 (PMC13434298; doi:10.1158/0008-5472.CAN-25-4426)
Supplement: Supplementary file 1 [file can-25-4426_supplemental_table_1_suppst1.docx]

**Supplemental Table 1. Opposing Roles of Glucocorticoid Receptor (GR) Signaling in Lymphoid Malignancies Versus Solid Tumors**

|  | **Lymphoid Malignancies ^1-3^** | **Solid Tumors ^4-11^** |
| --- | --- | --- |
| **Overall effect of GR activation** | Pro-apoptotic | Anti-apoptotic |
| **Key molecular changes** | GR activation induces intrinsic apoptosis (BIM upregulation, inhibition of oncogenic B cell receptor signaling by repression  of BCL-2 family survival proteins) | GR activation suppresses apoptotic signaling (upregulation of anti-apoptotic genes *SGK1* and *DUSP1*) |
| **Immune effects** | GR signaling causes mobilization/redistribution of malignant lymphocytes from protective niches (enhancing susceptibility to apoptosis) | Glucocorticoids exert immunosuppressive, anti-inflammatory effects and blunt anti-tumor immunity |
| **Clinical use/implications** | Glucocorticoids are essential disease-modifying therapy in ALL, CLL, and aggressive lymphomas; routinely used in cytotoxic chemotherapy regimens | Glucocorticoids are used as supportive care during cytotoxic therapy to reduce nausea, hypersensitivity, and treat immune-related adverse events; however, GR signaling reduces sensitivity to chemotherapy and immunotherapy |
| **Direction of therapeutic targeting** | Preserve or enhance GR signaling | Inhibit or antagonize GR signaling |

ALL, acute lymphoblastic leukemia; BCL-2, apoptosis regulator; BIM, BCL2 like 11; CLL, chronic lymphocytic leukemia; DUSP1, dual-specificity phosphatase 1; SGK1, serum/glucocorticoid-regulated kinase 1.

**References**

1. Burger JA, Montserrat E. Coming full circle: 70 years of chronic lymphocytic leukemia cell redistribution, from glucocorticoids to inhibitors of B-cell receptor signaling. *Blood* **2013**;121:1501–09.
2. Choi J, Ceribelli M, Phelan JD, Haupl B, Huang DW, Wright GW, *et al*. Molecular targets of glucocorticoids that elucidate their therapeutic efficacy in aggressive lymphomas. *Cancer Cell* **2024**;42:833–49.e12.
3. Inaba H, Pui CH. Glucocorticoid use in acute lymphoblastic leukaemia. *Lancet Oncol* **2010**;11:1096–106.
4. Melhem A, Yamada SD, Fleming GF, Delgado B, Brickley DR, Wu W, *et al*. Administration of glucocorticoids to ovarian cancer patients is associated with expression of the anti-apoptotic genes SGK1 and MKP1/DUSP1 in ovarian tissues. *Clin Cancer Res* **2009**;15:3196–204.
5. Kim D, Nguyen QT, Lee J, Lee SH, Janocha A, Kim S, *et al*. Anti-inflammatory roles of glucocorticoids are mediated by Foxp3+ regulatory T cells via a miR-342-dependent mechanism. *Immunity* **2020**;53:581–96.e5.
6. de Castro Baccarin AL, Irene MN, de Iracema Gomes Cubero D, Luz AS, Castro SN, Sordi R, *et al*. The feasibility of dexamethasone omission in weekly paclitaxel treatment for breast cancer patients. *Supportive Care Cancer* **2019**;27:927–31.
7. Kim KN, LaRiviere M, Macduffie E, White CA, Jordan-Luft MM, Anderson E, *et al*. Use of glucocorticoids in patients with cancer: potential benefits, harms, and practical considerations for clinical practice. *Pract Radiat Oncol* **2023**; 13:28–40.
8. Petrelli F, Bukovec R, Perego G, Luisa R, Luciani A, Zaniboni A, *et al*. Association of steroid use with survival in solid tumours. *Eur J Cancer* **2020**;141:105–14.
9. Greenstein AE, Hunt HJ. Glucocorticoid receptor antagonism promotes apoptosis in solid tumor cells. *Oncotarget* **2021**;12:1243–55.
10. Stringer-Reasor EM, Baker GM, Skor MN, Kocherginsky M, Lengyel E, Fleming GF, Conzen SD, *et al*. Glucocorticoid receptor activation inhibits chemotherapy-induced cell death in high-grade serous ovarian carcinoma. *Gynecol Oncol* **2015**;138:656–62.
11. Veneris JT, Darcy KM, Mhawech-Fauceglia P, Tian C, Lengyel E, Lastra RR, *et al*. High glucocorticoid receptor expression predicts short progression-free survival in ovarian cancer. *Gynecol Oncol* **2017**;146:153–60.
