# Supplementary material for: Signaling Under Stress: Targeting the Glucocorticoid Receptor in Cancer
Source: Cancer Res. 2026 May 18;86(15):3636–48. doi: 10.1158/0008-5472.CAN-25-4426 (PMC13434298; doi:10.1158/0008-5472.CAN-25-4426)
Supplement: Supplementary file 2 [file can-25-4426_supplemental_table_2_suppst2.docx]

**Supplemental Table 2. Glucocorticoid Receptor (GR) Antagonists Assessed in Patients With Solid Tumors**

| Compound | Mechanism | Clinical trial(s) | Tumor type(s) | Phase | Status |
| --- | --- | --- | --- | --- | --- |
| Relacorilant (CORT125134) | Selective GR antagonist | ROSELLA (NCT05257408)^1^: relacorilant + nab-paclitaxel | PROC | Phase 3 | Ongoing |
| *Nonsteroidal small molecule* |  | NCT03674814^2^: relacorilant + enzalutamide | mCRPC | Phase 1 | Ongoing |
|  |  | BELLA (NCT06906341)^3^: relacorilant + nab-paclitaxel +/- bevacizumab | PROC; PSOC; endometrial cancer | Phase 2 | Ongoing |
|  |  | NCT05726292^4^: relacorilant + enzalutamide | High-risk localized PC | Phase 2 | Ongoing |
|  |  | NCT07259317^5^: relacorilant +  nab-paclitaxel + gemcitabine | PDAC | Phase 2 | Ongoing |
| Exicorilant (CORT125281)  *Nonsteroidal small molecule* | Selective GR antagonist | NCT03437941^6^: exicorilant +  enzalutamide | mCRPC | Phase 1/2a | Completed |
| ORIC-101  *Steroidal small molecule* | Mifepristone analogue with improved GR selectivity | NCT03928314^7^: ORIC-101 +  nab-paclitaxel | Solid tumors | Phase 1 | Discontinued |
|  |  | NCT04033328^8^: ORIC-101 + enzalutamide | mCRPC | Phase 1b | Discontinued |
| Mifepristone  *Steroidal small molecule* | Nonselective GR antagonist (GR, PR and AR activity) | NCT03225547^9^: mifepristone + pembrolizumab | HER2-negative mBC | Phase 2 | Ongoing |
|  |  | NCT02014337^10^: mifepristone + eribulin | mTNBC | Phase 1 | Completed |
|  |  | NCT02012296^11^: mifepristone + enzalutamide | mCRPC | Phase 1/2 | Completed |

AR, androgen receptor; mBC, metastatic breast cancer; mCRPC, metastatic castration-resistant prostate cancer; mTNBC, metastatic triple-negative breast cancer; PC, prostate cancer; PDAC, pancreatic ductal adenocarcinoma; PR, progesterone receptor; PROC, platinum-resistant ovarian cancer; PSOC, platinum-sensitive ovarian cancer.

**References**

1. ClinicalTrials.gov. Relacorilant in combination with nab-paclitaxel in advanced, platinum-resistant, high-grade epithelial ovarian, primary peritoneal, or fallopian-tube cancer. Identifier: NCT05257408. Bethesda (MD): National Library of Medicine. Available from: https://clinicaltrials.gov/study/NCT05257408.
2. ClinicalTrials.gov. Study of drug 1 (enzalutamide) plus drug 2 (relacorilant) for patients with prostate cancer. Identifier: NCT03674814. Bethesda (MD): National Library of Medicine. Available from: https://clinicaltrials.gov/study/NCT03674814.
3. ClinicalTrials.gov**.** Relacorilant in combination with different treatment regimens in patients with gynecological cancers. Identifier: NCT06906341. Bethesda (MD): National Library of Medicine. Available from: https://clinicaltrials.gov/study/NCT06906341.
4. ClinicalTrials.gov**.** A study of enzalutamide plus the glucocorticoid receptor antagonist relacorilant versus placebo for patients with high-risk localized prostate cancer. Identifier: NCT05726292. Bethesda (MD): National Library of Medicine. Available from: https://clinicaltrials.gov/study/NCT05726292.
5. ClinicalTrials.gov**.** Relacorilant with nab-paclitaxel and gemcitabine in patients with metastatic pancreatic adenocarcinoma. Identifier: NCT07259317. Bethesda (MD): National Library of Medicine. Available from: https://clinicaltrials.gov/study/NCT07259317.
6. ClinicalTrials.gov**.** Study to evaluate CORT125281 in combination with enzalutamide in patients with mCRPC. Identifier: NCT03437941. Bethesda (MD): National Library of Medicine. Available from: https://clinicaltrials.gov/study/NCT03437941.
7. ClinicalTrials.gov**.** Study of ORIC-101 in combination with anticancer therapy. Identifier: NCT03928314. Bethesda (MD): National Library of Medicine. Available from: https://clinicaltrials.gov/study/NCT03928314.
8. ClinicalTrials.gov**.** Study of ORIC-101 in combination with enzalutamide. Identifier: NCT04033328. Bethesda (MD): National Library of Medicine. Available from: https://clinicaltrials.gov/study/NCT04033328.
9. ClinicalTrials.gov**.** Study of pembrolizumab and mifepristone in patients with advanced HER2-negative breast cancer. Identifier: NCT03225547. Bethesda (MD): National Library of Medicine. Available from: https://clinicaltrials.gov/study/NCT03225547.
10. ClinicalTrials.gov**.** [Mifepristone and eribulin in patients with metastatic triple negative breast cancer or other specified solid tumors](https://clinicaltrials.gov/study/NCT02014337?term=NCT02014337&rank=1). Identifier: NCT02014337. Bethesda (MD): National Library of Medicine. Available from: https://clinicaltrials.gov/study/NCT02014337.
11. ClinicalTrials.gov**.** Enzalutamide and mifepristone in treating patients with metastatic hormone resistant prostate cancer. Identifier: NCT02012296. Bethesda (MD): National Library of Medicine. Available from: https://clinicaltrials.gov/study/NCT02012296.
